# Cell foundry with high product specificity and catalytic activity for 21-deoxycortisol biotransformation

**DOI:** 10.1186/s12934-017-0720-y

**Published:** 2017-06-13

**Authors:** Shuting Xiong, Ying Wang, Mingdong Yao, Hong Liu, Xiao Zhou, Wenhai Xiao, Yingjin Yuan

**Affiliations:** 1 Key Laboratory of Systems Bioengineering (Ministry of Education), Tianjin University, No. 92, Weijin Road, Nankai District, Tianjin, 300072 People’s Republic of China; 2 SynBio Research Platform, Collaborative Innovation Center of Chemical Science and Engineering (Tianjin), School of Chemical Engineering and Technology, Tianjin University, Tianjin, 300072 People’s Republic of China

**Keywords:** Synthetic biology, Whole-cell biocatalysis, Product specificity, Catalytic activity, 21-Deoxycortisol, CYP11B1

## Abstract

**Background:**

21-deoxycortisol (21-DF) is the key intermediate to manufacture pharmaceutical glucocorticoids. Recently, a Japan patent has realized 21-DF production via biotransformation of 17-hydroxyprogesterone (17-OHP) by purified steroid 11β-hydroxylase CYP11B1. Due to the less costs on enzyme isolation, purification and stabilization as well as cofactors supply, whole-cell should be preferentially employed as the biocatalyst over purified enzymes. No reports as so far have demonstrated a whole-cell system to produce 21-DF. Therefore, this study aimed to establish a whole-cell biocatalyst to achieve 21-DF transformation with high catalytic activity and product specificity.

**Results:**

In this study, *Escherichia coli* MG1655(DE3), which exhibited the highest substrate transportation rate among other tested chassises, was employed as the host cell to construct our biocatalyst by co-expressing heterologous CYP11B1 together with bovine adrenodoxin and adrenodoxin reductase. Through screening CYP11B1s (with mutagenesis at N-terminus) from nine sources, *Homo sapiens* CYP11B1 mutant (G25R/G46R/L52 M) achieved the highest 21-DF transformation rate at 10.6 mg/L/h. Furthermore, an optimal substrate concentration of 2.4 g/L and a corresponding transformation rate of 16.2 mg/L/h were obtained by screening substrate concentrations. To be noted, based on structural analysis of the enzyme-substrate complex, two types of site-directed mutations were designed to adjust the relative position between the catalytic active site heme and the substrate. Accordingly, 1.96-fold enhancement on 21-DF transformation rate (to 47.9 mg/L/h) and 2.78-fold improvement on product/by-product ratio (from 0.36 to 1.36) were achieved by the combined mutagenesis of F381A/L382S/I488L. Eventually, after 38-h biotransformation in shake-flask, the production of 21-DF reached to 1.42 g/L with a yield of 52.7%, which is the highest 21-DF production as known.

**Conclusions:**

Heterologous CYP11B1 was manipulated to construct *E. coli* biocatalyst converting 17-OHP to 21-DF. Through the strategies in terms of (1) screening enzymes (with N-terminal mutagenesis) sources, (2) optimizing substrate concentration, and most importantly (3) rational design novel mutants aided by structural analysis, the 21-DF transformation rate was stepwise improved by 19.5-fold along with 4.67-fold increase on the product/byproduct ratio. Eventually, the highest 21-DF reported production was achieved in shake-flask after 38-h biotransformation. This study highlighted above described methods to obtain a high efficient and specific biocatalyst for the desired biotransformation.

**Electronic supplementary material:**

The online version of this article (doi:10.1186/s12934-017-0720-y) contains supplementary material, which is available to authorized users.

## Background

**Fig. 1:**
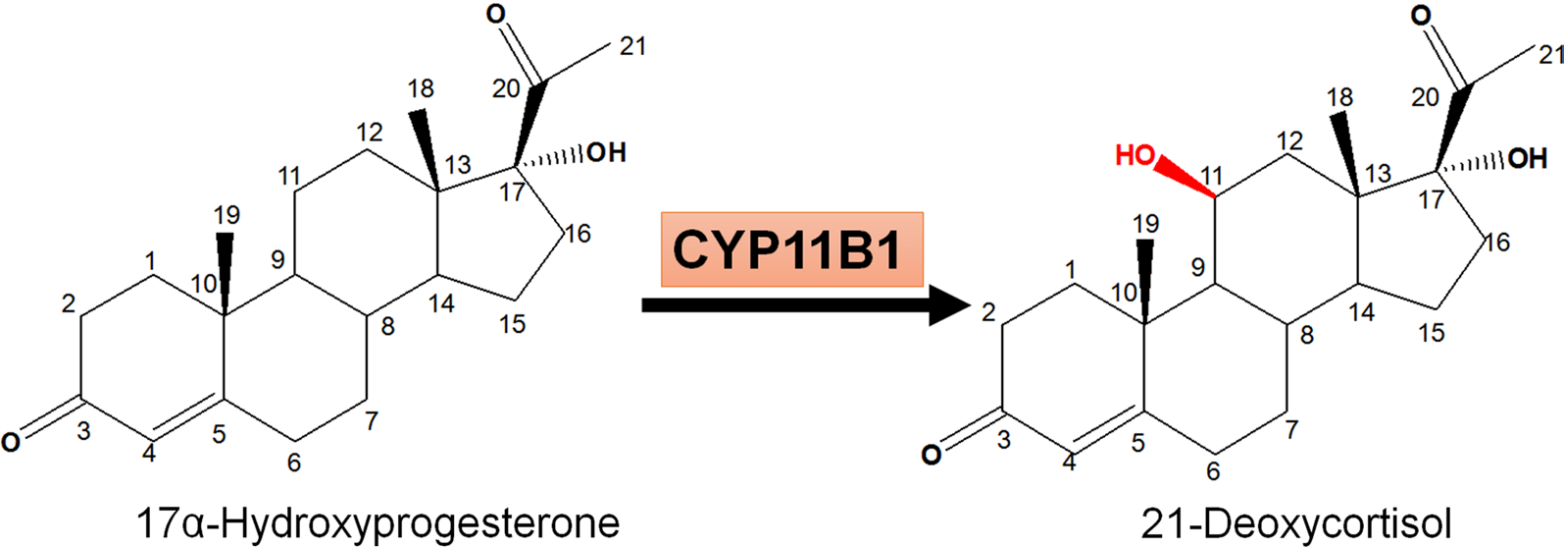
The paradigm of 21-deoxycortisol (21-DF) biosynthetic pathway from 17α-hydroxyprogesterone (17-OHP). CYP11B1, which is responsible for 11β-hydroxylation, was highlighted in *red*

Manufacturing steroid compounds with therapeutic usage and commercial value is a successful example to apply biocatalysts in large-scale industrial processes [1–4]. The high regio- and stereo-selectivity make bioconversion more advantageous than chemical synthesis of steroid molecules with complex structures [5, 6]. Considering the costs involved in enzyme isolation, purification and stabilization as well as cofactors [such as NAD(P)H] supply, whole-cell as biocatalysts is better than purified enzyme to some extent [5, 6]. As one of steroids, 21-deoxycortisol (21-DF) is the key intermediate for pharmaceutical glucocorticoids with anti-inflammatory effect. For instance, it can be used as the substrate to chemosynthesize cortisol via a matured and environmental friendly process [7]. However, the accessibility of 21-DF in bulks remains a great challenge due to catalytic selectivity for 11β-hydroxylation, which has restricted the large-scale application of 21-DF in associated steroid industry. In 2006, a Japan patent [8] realized 21-DF transformation only through one step catalysis by purified adrenodoxin (Adx), adrenodoxin reductase (AdR) and CYP11B1. However, no reports as known has demonstrated a whole-cell system for 21-DF transformation. Therefore, this study aims to establish a whole-cell biocatalyst of CYP11B1 to achieve the desired conversion (Fig. 1) with high catalytic activity and product specificity.

Cytochrome P450s are highly involved in biocatalysts [9–16], catalyzing a broad range of reactions including steroid hydroxylation [1, 17–20]. For manipulation of heterologous P450 in an appropriate host cell, many approaches have been successfully applied to improve the catalytic activity and soluble expression level of the target protein [9, 21–25]. And some P450s exhibiting relaxed substrate specificities [26–29], probably leading to a concerted oxidation reaction without structural selectivity [30]. Therefore, poor product specificity brought by these P450s became a main obstacle [31, 32]. In order to alleviate by-product accumulation, site-direct mutagenesis within P450s was applied to alter product profiles, in which those successful mutations were obtained in irrational [33–35], semi-rational [27, 36–38] or even rational design [39–41]. Compared with other approaches, rational design not only avoids laborious and time-consuming operation, but also directly builds up the correlation between mutant structures and catalytic mechanism. Majority of the hot spot positions for site-directed mutagenesis were within or surround the active site cavity [39–41], which aimed to make relatively minor alternations on substrate-recognition sites and substrate-access channels to strengthen the targeted substrate binding and reposition the compound of interest [37, 42]. In order to increase the proportion of perillyl alcohol, Seifert et al. [43] once designed mutations to reshape the substrate binding cavity of CYP102A1 by selectively exposing the C7 of (4R)-limonene towards the activated heme oxygen. For our targeted protein CYP11B1, Schiffer et al. [44] once reported that CYP11B1-dependent biocatalyst transformed spironolactone into three isomers with hydroxyl group at C11, C18 and C19, respectively, which might due to the relative position between the potential hydroxylation sites and the catalytic active site heme. Thus, in order to concentrate our target product 21-DF, it will be promising solution to regulate the relative position between the substrate and the catalytic active site heme through the mutagenesis especially at essential residues associated to heme.

In this study, a whole-cell biocatalyst of CYP11B1 was established to transform 17-OHP into 21-DF. Through screening five host candidates with unique characteristics to fit steroid conversion, *E. coli* MG1655(DE3), which exhibited the highest substrate transportation rate, was employed as the host cell to construct our biocatalyst. The key enzyme CYP11B1 has already been successfully expressed in both eukaryotes (such as *Saccharomyces cerevisiae* [45] and *Schizosaccharomyces pombe* [46]) and prokaryotes (such as *E. coli* [11]) to achieve the conversion of 11-deoxycortisol to cortisol, those approaches to improve the expression level and catalytic activity of CYP11B1 would provide valuable information to assist our work. Notably, in order to improve the catalytic activity as well as product specificity of our biocatalyst, a series of novel mutations were designed based on structural analysis of the enzyme-substrate complex to regulate the relative position between the substrate 17-OHP and the catalytic active site heme in CYP11B1. 19.5-fold increase on 21-DF transformation rate along with 4.67-fold enhancement on product/by-product ratio were totally achieved via screening CYP11B1s from diverse sources, N-terminal mutagenesis, optimizing substrate concentration and rational design site-directed mutations in the activity pocket of CYP11B1. Eventually, the production of 21-DF reached to 1.42 g/L with a yield of 52.7%, which is the highest 21-DF production as known. This study not only provides promising methods to optimize heterologous P450s by rational design, but also sets a good example of manipulation the key enzyme to systematically improve the efficiency and catalytic selectivity of the whole-cell biocatalyst to accomplish the desired reaction.

## Methods

### Strains and cultivation

*Escherichia coli* DH5α was used to construct and maintain plasmids, while *E. coli* C43(DE3) [47] and MG1655(DE3) [48] were attempted to be employed for steroids conversion. All the *E. coli* strains were cultured in Luria–Bertani (LB) medium at 37 °C. When needed, 100 μg/mL ampicillin and 34 μg/mL chloramphenicol were supplemented into the medium. In the meanwhile, three model organisms, i.e. *S. cerevisiae* CEN.PK2, *Yarrowia lipolytica* ATCC201249 [49] and *Mycobacterium smegmatis* mc^2^155 [50] were also attempted to convert steroids. *S. cerevisiae* and *Y. lipolytica* strains were grown in YPD medium [51] at 30 °C, while *M. smegmatis* strain was cultured in Middlebrook 7H9 broth (Difco) [52] at 37 °C.

### Plasmids construction and mutagenesis

Primers and plasmids used in this study were listed in Additional file 1: Tables S1 and S2, respectively. All the heterologous genes including *Cyp11B1*, *AdR*, and truncated *Adx* (*Adx*_*4*–*108*_) were codon optimized (Additional file 1: Table S3) and synthesized by GENEWIZ (Suzhou, China). These genes were delivered as pUC57-simple serious plasmids (Additional file 1: Table S2). All the Adx-CPY11B1-AdR expression plasmids were constructed based on vector pET-21a-YX, which was derived from vector pET-21a (Novagen, Germany) by Cao et al. [53] through adding a SpeI site between the BamHI and BglI site of the initial plasmid. Therefore, genes *Cyp11B1*, *Adx*_*4*–*108*_ and *AdR* could be assembled into pET-21a-YX via BioBrick™ strategy [54]. To be noted, as shown in Additional file 1: Figure S1, genes *Adx*_*4*–*108*_, *AdR* and *Cyp11B1* were recovered from pUC57-simple series plasmids by NdeI/SpeI digestion and inserted into the same sites of pET21a-YX, obtaining pXST-01 (pET21a-*Adx*_*4*–*108*_), pXST-02 (pET21a-*AdR*) and pXST-03–11 (pET21a-*Cyp11B1*), respectively. Then gene *Cyp11B1* was cut from the corresponding pET21a-*Cyp11B1* plasmids by XbaI/BamHI and inserted into the BamHI/SpeI sites of pXST-01, generating plasmids pXST-21–29 (pET21a-*Adx*_*4*–*108*_-*Cyp11B1*). After that, gene *AdR* was cut from plasmid pXST-02 by XbaI/BamHI and inserted into the BamHI/SpeI sites of plasmids pET21a-*Adx*_*4*–*108*_-*Cyp11B1* to construct the final Adx_*4*–*108*_-CPY11B1-AdR co-expression plasmids (pXST-39–47, Table 1) for steroids conversion.

**Table 1:** Strains used in this study

Strain	Description	Source
*E. coli* C43(DE3)	*F* ^−^ *ompT gal hsdSB (rB*- *mB*-*) dcm Ion λ*	[47]
*E. coli* MG1655(DE3)	MG1655 *ΔendA ΔrecA* (DE3), transformation strain	[48]
*S. cerevisiae*	MATα, *his3Δ1 leu2Δ0 lys2Δ0 ura3Δ0*	This lab
*Y. lipolytica*	MATA, *ura3*-*302, leu2*-*270, lys8*-*11, pex17*-*ha*	[49]
*M. smegmatis* mc^2^155	*ept*-*1 mc26* mutant efficient for electroporation	[50]
Ec02040101	*E. coli* MG1655(DE3) with plasmids pETXST39 (pET21a-*Adx*_*4*–*108*_-*Cyp11B1_*Hs-*AdR*) and pGro7	This study
Ec02040102	*E. coli* MG1655(DE3) with plasmids pETXST40 (pET21a-*Adx*_*4*–*108*_-*Cyp11B1*_Bt-*AdR*) and pGro7	This study
Ec02040103	*E. coli* MG1655(DE3) with plasmids pETXST41 (pET21a-*Adx*_*4*–*108*_-*Cyp11B1*_Rn-*AdR*) and pGro7	This study
Ec02040104	*E. coli* MG1655(DE3) with plasmids pETXST42 (pET21a-*Adx*_*4*–*108*_-*Cyp11B1_Oa*-*AdR*) and pGro7	This study
Ec02040105	*E. coli* MG1655(DE3) with plasmids pETXST43 (pET21a-*Adx*_*4*–*108*_-*Cyp11B1_Sh*-*AdR*) and pGro7	This study
Ec02040106	*E. coli* MG1655(DE3) with plasmids pETXST44 (pET21a-*Adx*_*4*–*108*_-*Cyp11B1_Pa*-*AdR*) and pGro7	This study
Ec02040107	*E. coli* MG1655(DE3) with plasmids pETXST45 (pET21a-*Adx*_*4*–*108*_-*Cyp11B1_Cco*-*AdR*) and pGro7	This study
Ec02040108	*E. coli* MG1655(DE3) with plasmids pETXST46 (pET21a-*Adx*_*4*–*108*_-*Cyp11B1_Mo*-*AdR*) and pGro7	This study
Ec02040109	*E. coli* MG1655(DE3) with plasmids pETXST47 (pET21a-*Adx*_*4*–*108*_-*Cyp11B1_Sa*-*AdR*) and pGro7	This study
Ec02040110	*E. coli* MG1655(DE3) with plasmids pETXST48 (pET21a-*Adx*_*4*–*108*_-*Cyp11B1_*Hs_G25R/G46R/L52M_-*AdR*) and pGro7	This study
Ec02040111	*E. coli* MG1655(DE3) with plasmids pETXST49 (pET21a-*Adx*_*4*–*108*_-*Cyp11B1*_Bt_G25R_-*AdR*) and pGro7	This study
Ec02040112	*E. coli* MG1655(DE3) with plasmids pETXST50 (pET21a-*Adx*_*4*–*108*_-*Cyp11B1*_Rn_G25R/G46R_-*AdR*) and pGro7	This study
Ec02040113	*E. coli* MG1655(DE3) with plasmids pETXST51 (pET21a-*Adx*_*4*–*108*_-*Cyp11B1*_Oa_G25R/G46R/V52M_-*AdR*) and pGro7	This study
Ec02040114	*E. coli* MG1655(DE3) with plasmids pETXST52 (pET21a-*Adx*_*4*–*108*_-*Cyp11B1*_Sh_T26R/S55R_-*AdR*) and pGro7	This study
Ec02040115	*E. coli* MG1655(DE3) with plasmids pETXST53 (pET21a-*Adx*_*4*–*108*_-*Cyp11B1*_Pa_G25R/G46R/A52M_-*AdR*) and pGro7	This study
Ec02040116	*E. coli* MG1655(DE3) with plasmids pETXST54 (pET21a-*Adx*_*4*–*108*_-*Cyp11B1*_Cco_Y20R/W38R/N44M_-*AdR*) and pGro7	This study
Ec02040117	*E. coli* MG1655(DE3) with plasmids pETXST55 (pET21a-*Adx*_*4*–*108*_-*Cyp11B1*_Mo_A30R/S50R/D56M_-*AdR*) and pGro7	This study
Ec02040118	*E. coli* MG1655(DE3) with plasmids pETXST56 (pET21a-*Adx*_*4*–*108*_-*Cyp11B1*_Sa_A30R/V41R/L47M_-*AdR*) and pGro7	This study
Ec02040119	*E. coli* MG1655(DE3) with plasmids pETXST57 (pET21a*Adx*_*4*–*108*_-*Cyp11B1_*Hs_G25R/G46R/L52M/R384A_-*AdR*) and pGro7	This study
Ec02040120	*E. coli* MG1655(DE3) with plasmids pETXST58 (pET21a- *Adx*_*4*–*108*_-*Cyp11B1_*Hs _G25R/G46R/L52M/R110A_-*AdR*) and pGro7	This study
Ec02040122	*E. coli* MG1655(DE3) with plasmids pETXST59 (pET21a-*Adx*_*4*–*108*_-*Cyp11B1_*Hs _G25R/G46R/L52M/F381A/L382S_-*AdR*) and pGro7	This study
Ec02040123	*E. coli* MG1655(DE3) with plasmids pETXST60 (pET21a-*Adx*_*4*–*108*_-*Cyp11B1_*Hs _G25R/G46R/L52M/F381A/L382T_-*AdR*) and pGro7	This study
Ec02040124	*E. coli* MG1655(DE3) with plasmids pETXST61 (pET21a-*Adx*_*4*–*108*_-*Cyp11B1_*Hs _G25R/G46R/L52M/I488L_-*AdR*) and pGro7	This study
Ec02040125	*E. coli* MG1655(DE3) with plasmids pETXST62 (pET21a-*Adx*_*4*–*108*_-*Cyp11B1_*Hs _G25R/G46R/L52M/F381A/L382S/I488L_-*AdR*) and pGro7	This study

A modified overlap extension PCR (OE-PCR) was employed for mutagenesis in CYP11B1. As illustrated in Additional file 1: Figure S2, mutated residue(s) were introduced into the tails of matched primers OE-1 and OE-2, while NdeI and SpeI restriction sties were introduced into 5′ end of primers OE-F and OE-R, respectively. The products of the first round of PCR with primer pairs OE-F/OE-1 and OE-2/OE-R separately were used as the templates of the second round of PCR with primer pairs OE-F/OE-R. Then the final amplification product was digested by NdeI/SpeI for constructing the final Adx-CPY11B1-AdR co-expression plasmid via the method described above.

### Substrate feeding assay

The substrate-feeding assay was carried out in 250 mL flasks with 50 mL appropriate medium according to organism specie. The substrate 17-OHP was supplemented into the culture at the very beginning of the cultivation from a stock solution in 50% (m/v) (2-Hydroxypropyl)-γ-cyclodextrin. The concentration of 17-OHP was 36 mg/mL in the stock solution and diluted to 720 μg/mL in final reaction solution. During this experiment, samples were taken every 8 h.

### Whole-cell biocatalysis in shake-flask

In this study, whole-cell biocatalysis was separated into two periods as protein expression and steroid conversion, respectively. All the experiments involving in these two processes were conducted by *E. coli* MG1655(DE3). This strain was kindly provided by Professor Kristala L. J. Prather from Massachusetts Institute of Technology (MIT), USA. Before the whole-cell biocatalysis, the particular Adx-CPY11B1-AdR co-expression plasmid together with chaperone vector pGro7 (Takara, Japan) were transformed into *E. coli* MG1655(DE3) by electroporation. Plasmid pGro7 contains two chaperone genes *groEL* and *groES* to assist protein folding [55]. Then the recombinant strains were selected from LB agar plates supplemented with 100 μg/mL ampicillin and 34 μg/mL chloramphenicol.

The procedures for protein expression and steroids conversion were modified according to Schiffer et al. [11]. For protein expression, the recombinant strains for whole-cell biocatalysis were grown in 4 mL TB medium (12 g/L peptone, 24 g/L yeast extract, 0.4% (v/v) glycerol, 4.62 g/L KH_2_PO_4_, 25 g/L K_2_HPO_4_) with 100 μg/mL ampicillin and 34 μg/mL chloramphenicol, shaking at 37 °C, 250 rpm overnight. After that, the preculture was transferred into 50 mL fresh TB medium with the same antibiotics and grown at 37 °C, 250 rpm until the OD_600_ reached 0.5–0.7. Then the temperature and shaking speed were reduced to 25 °C and 200 rpm, respectively. After that 1 mM IPTG, 1 mM the heme precursor δ-aminolevulinic acid, 4 mg/mL l-arabinose and 50 μg/mL ampicillin were added into the medium to initialize protein expression. The protein expression period would last for 21 h.

For steroids conversion with resting cells, the cultures from protein expression process were harvested by centrifugation (8000 rpm, at 4 °C for 5 min). Then the cells were washed twice by 50 mM potassium phosphate buffer (pH 7.4) and resuspended by the steroids conversion solution containing 50 mM potassium phosphate buffer (pH 7.4), 2% (v/v) glycerol, 1 mM IPTG, 1 mM δ-aminolevulinic acid, 4 mg/mL l-arabinose, 100 μg/mL ampicillin and 34 μg/mL chloramphenicol. The cell density (OD_600_) in this solution maintained in 75–85. The substrate 17-OHP was supplemented into the solution with appropriate concentration. Biotransformation was performed at 27.5 °C. Samples were taken at the defined time points. According to the data in Additional file 1: Figures S4, S5a and S6, the data at 12 and 18 h were all in the linear range of all the conversion. Therefore, the maximal conversion rate (mg/L/h) was determined as (P_18_–P_12_)/(t_18_–t_12_), where P is for product (21-DF) concentration (mg/L) and t is for time (h).

### Extraction and analysis of steroids

The standards of 17-OHP and 21-DF were purchased from Sigma (Sigma-Aldrich, USA). The procedures for extracting and analyzing the related steroids were modified according to the methods for 11-deoxycortisol and cortisol [11]. To be specific, 10 mL samples were extracted by 10 mL dichloromethane twice. After centrifugation at 8000 rpm for 5 min, the organic solvent containing steroids on the bottom was collected and then evaporated by nitrogen blow. Then products were dissolved in methanol and analyzed by high-performance liquid chromatography system (HPLC, Waterse2695, Waters Corp., USA) equipped with a BDS HYPERSIL C18 column (150 mm × 4.6 mm, 5 μm, Thermo Scientific) and a Photodiode array detector (Waters 2996). The signals were detected at 240 nm. 70% (v/v) methanol–water (containing 0.1% formic acid) was chosen as the mobile phase with a flow rate at 1 mL/min and the column temperature was set at 40 °C. Semi-quantitative analysis of the unidentified by-product was applied based on the standard curve of 21-DF.

### Bioinformatics and structural analysis of CYP11B1

The amino acid sequences of CYP11B1 from diversity species were aligned by ClustalW with default settings [56]. In the meanwhile, in order to analyze and design the complex structure of CYP11B1_Hs mutant1 (G25R/G46R/L52M) with the substrate, three-dimensional structure models were developed by Swiss-Model (http://swissmodel.expasy.org/) with the high sequence homology (>90% identify) and high-resolution crystal structure of CYP11B2 (the aldosterone synthase from *Homo sapiens* with ligand, PDB accession 4dvq-F) as the template. The structure models were subjected to energy minimization by Swiss-Pdb Viewer (http://spdbv.vital-it.ch/). Afterwards enzyme-ligand docking was performed by AutoDockVina program [57]. And the docking studies were run with 17-OHP as the ligand for above-mentioned structure model. The 17-OHP structure files (ligand) were retrieved from ZINC site [58]. Docking cluster analysis was performed in the AutoDockVina program environment, and clusters were characterized by binding energy (in kilocalories per mole). Establishment of docking models was also followed by energy minimization. The built complex structural analysis was done with Pymol software [59]. The mutation at specific amino acid site was also introduced by this software, which allowed exploration of the spatial and molecular interactions among amino acids.

## Results and discussion

### Selected the host cell with the strongest capability of substrate transportation

**Fig. 2:**
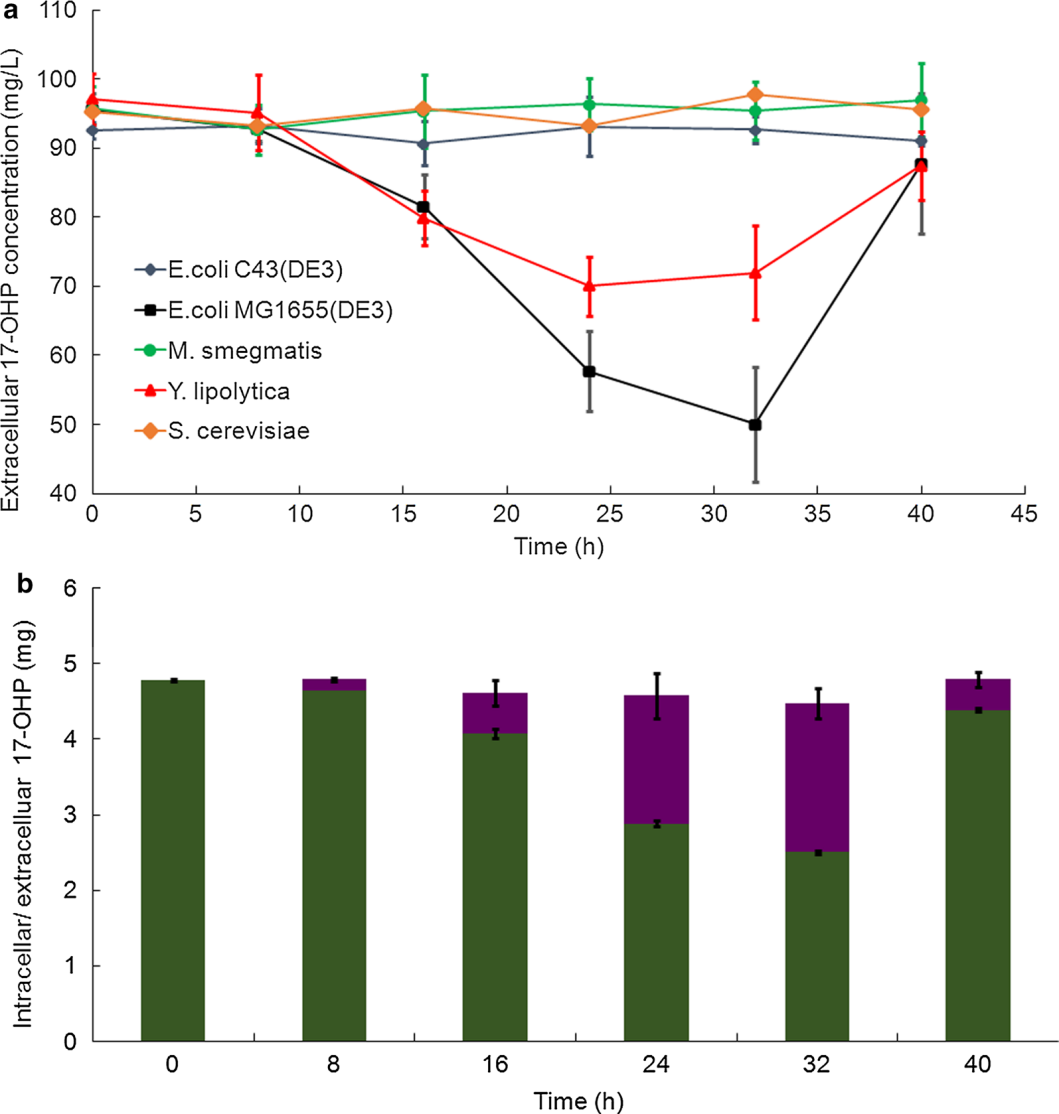
Selecting the host cell processing high substrate transportation ability. **a** Extracellular substrate (17-OHP) concentration for *E. coli* C43(DE3) (*dark blue*), *E. coli* MG1655(DE3) (*black*), *S. cerevisiae* CEN.PK2 (*orange*), *Y. lipolytica* ATCC201249 (*red*) and *M. smegmatis* mc^2^155 (*green*) over the time course. **b** The intracelluar (*purple*) and extracelluar (*green*) substrate amounts for *E. coli* MG1655(DE3) over the time course. The substrate 17-OHP was supplemented into the culture at the very beginning of the cultivation. Samples were taken every 8 h

Engineered yeast and *E. coli* strains have been reported to realize the conversion of 11-deoxycortisol to cortisol by CYP11B1 [60]. Meanwhile, *Y. lipolytica* strains are good at absorbing hydrophobic materials as well as accumulating lipid bodies that can store less polar metabolites and avoid cell burden [61]. *Mycobacterium* strains were widely applied to convert steroid compounds in pharmaceutical industry. In sum, the strains belonging to these species process distinguishing characteristics that might suit our desired biocatalyst for 21-DF production. In this study, the model organism from these species, i.e. *E. coli* MG1655(DE3), *E. coli* C43(DE3), *S. cerevisiae* CEN.PK2, *Y. lipolytica* ATCC201349 and *M. smegmatis* mc^2^155, were selected and their transportion capabilities of the substrate 17-OHP across the biocatalyst membrane were initially characterized. Through measuring the extracellular 17-OHP concentration at each time point, it was demonstrated that 17-OHP concentration was decreased significantly only in the media culturing *Y. lipolytica* ATCC201249 or *E. coli* MG1655(DE3) (Fig. 2a). And the most dramatic decline in extracellular 17-OHP concentration was found in the medium with *E. coli* MG1655(DE3) (Fig. 2b), indicating this strain exhibited the strongest ability to transport 17-OHP. As previous study demonstrated that endogenous protein GCY1 could convert 17-OHP into 17α,20α-dihydroxypregn-4-ene-3-one in *S. cerevisiae* [45], and *E. coli* protein Akr is the isoenzyme of GCY1 (http://www.kegg.jp). Therefore, whether the substrate 17-OHP was metabolized in *E. coli* MG1655(DE3) should be investigated. As shown in Fig. 2b, it was observed that the sums of extracellular and intracelluar 17-OHP amount were almost equal at each time point during the cultivation of *E. coli* MG1655(DE3), suggesting 17-OHP is stable during this period and strain MG1655(DE3) could not metabolize 17-OHP. Thus, *E. coli* MG1655(DE3) was employed as the host cell to construct the whole-cell biocatalysis in this study.

### Construct of a *E. coli* biocatalyst to convert 17-OHP into 21-DF

**Fig. 3:**
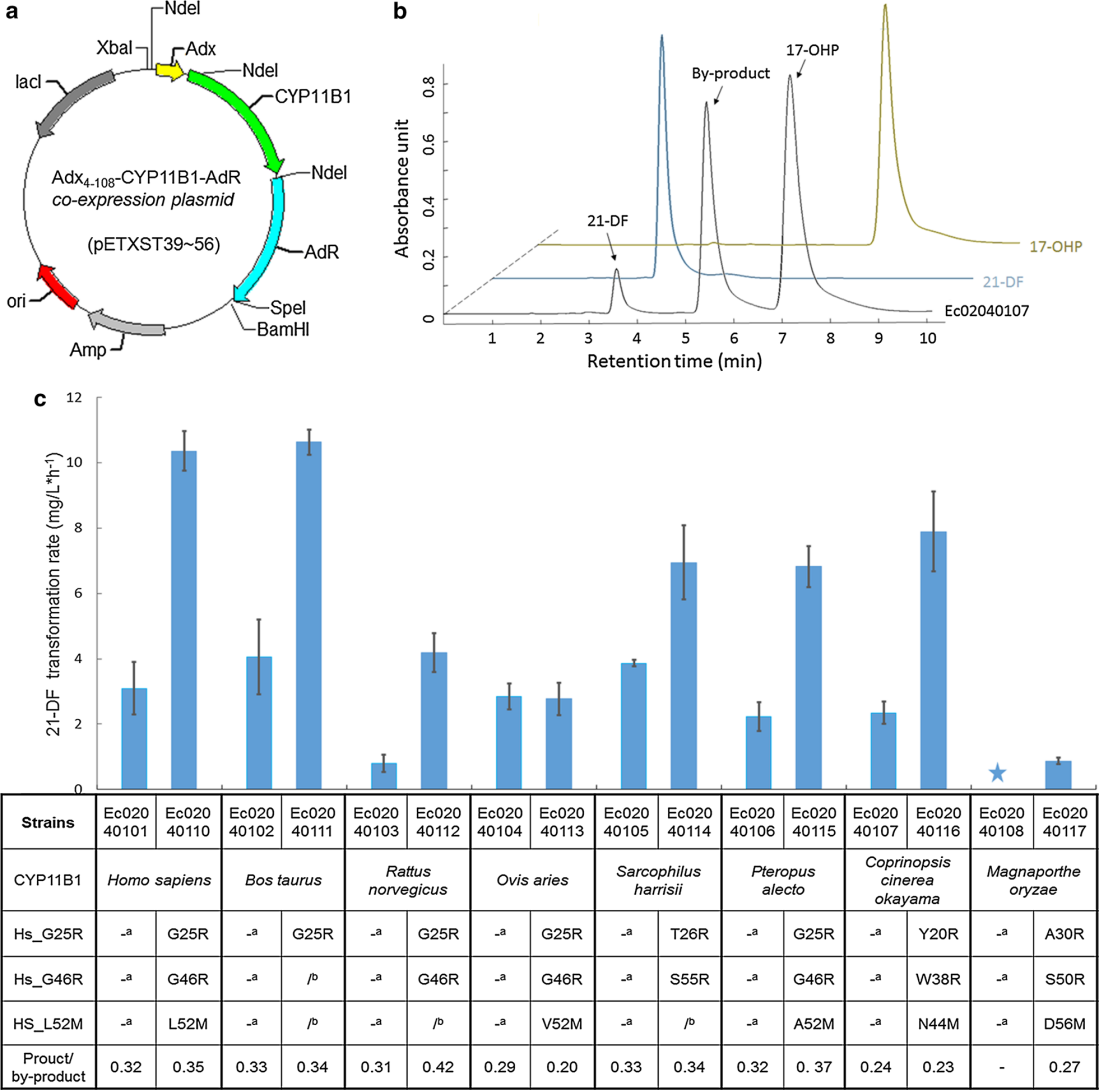
Construction of the whole-cell biocatalyst and improvement of its efficiency via screening CYP11B1 sources from the wild-type enzymes and N-terminal mutants. **a** The sketch of Adx_*4*–*108*_-CPY11B1-AdR co-expression plasmids (pETXST39–56) for steroids conversion. **b** The HPLC profile of strain EC02040107 (*black*) as well as the standard of 21-DF (*blue*) and 17-OHP (*yellow*). The signals of 21-DF, 17-OPH and the by-product were detected at 240 nm. **c** 21-DF transformation rates in shake flask fermentation. Strains EC02040101–08 carried wild-type CYP11B1s from diversity species, while strains EC02040110–17 harbored different CYP11B1s with mutated N-terminus. The mutagenesis, which were equal to the mutations reported in *H. sapiens* (Hs_G25R, Hs_G46R and Hs_L52M), were listed below their corresponding strain names. Symbol “-” was for unmutated residue, and symbol “/” suggested this mutation has existed in the associated enzymes. In the meanwhile, no 21-DF has been detected in the product of strain EC02040108, which was marked by *blue star*

In order to realize the reaction from 17-OHP to 21-DF by biocatalyst, heterologous CYP11B1 as well as its redox patterns (Adx and AdR) from *Bos taurus* were all codon optimized and introduced into *E. coli* MG1655(DE3). Adx was truncated from R1 to T3 (Adx4–108) to improve the efficiency of electron transfer [62]. As illustrated in Fig. 3a, all these three encoding genes were assembled into one cluster and carried by a T7 RNA polymerase-based vector. According to Nishihara et al. [63], bacterial chaperonin GroEL and its cofactor GroES, were also introduced into this system and co-expressed with the heterologous proteins to ensure proper folding. CYP11B1 from *Coprinopsis cinerea okayama* (CYP11B1_Cco), was initially chosen to establish the whole-cell system, generating strain Ec02040107. According to Schiffer et al. [11], the whole-cell biocatalysis procedure was separated into two periods as protein expression and steroid conversion, respectively. Transformation of 17-OHP was conducted with resting cells to avoid indole accumulation under glucose depletion [64], since indole might inhibit the activity of P450 [65]. The extract from the steroids conversion solution with strain Ec02040107 was analyzed by HPLC. As shown in Fig. 3b, 45.3 mg/L 21-DF (at 3.5 min, the same retention time as 21-DF standard) was successfully detected after 24-h transformation, indicating conversion of 17-OHP to 21-DF was succeeded here. However, there was plenty of the substrate (17-OHP, at 7.0 min) remaining here, and one kind of unidentified by-product (5.5 min) accumulated dramatically in the extracts (Fig. 3b).

### Improve biocatalyst efficiency via screening CYP11B1 sources, N-terminal mutagenesis and optimizing substrate concentration

Screening enzymes from diverse sources has been wildly applied and proved to be a promising strategy to overproduce desired compounds from single carbon sources [66–70]. Here, this method was also used to improve the conversion rate of our biocatalyst. Besides CYP11B1_Cco, eight other wild-type CYP11B1s from *H. sapiens* (CYP11B1_Hs), *Bos taurus* (CYP11B1_Bt), *Rattus norvegicus* (CYP11B1-_Rn), *Ovis aries* (CYP11B1_Oa), *Sarcophilus harrisii* (CYP11B1_Sh), *Pteropus alecto* (CYP11B1_Pa), *Magnaporthe oryzae* (CYP11B1_Mo) and *Sorex araneus* (CYP11B1_Sa) were selected to construct the whole-cell system accordingly, obtaining strains Ec02040101–09. And N-terminus modification (drastic truncation or site-directed mutagenesis) in P450s can impact their catalytic activity as well as expression level [71]. Schiffer et al. [11] once reported that mutagenesis at G23R of CYP11B1_Hs increased its expression level as well as maintained its catalytic activity for the reaction from 11-deoxycortisol to cortisol in *E. coli*. It was also reported mutations G46R [11, 72] and L52M [46] could enhance the activity of CYP11B1_Hs. Since mutations effected in one enzyme can be transposed to its homologous proteins [40], therefore these mutations were also introduced into all the CYP11B1s selected in this study. By aligning the protein sequences of these enzymes, the amino acid residues equal to G23, G46 and L52 within CYP11B1_Hs were identified in all the selected CYP11B1 (Additional file 1: Figure S3a) and then were mutated accordingly, obtaining strains Ec02040110–18. Strains Ec02040101–09 containing the wild-type CYP11B1s and strains Ec02040110–18 harboring the mutated CYP11B1s from diverse species were cultured in shake flask to determine the corresponding transformation rate for 21-DF. As a result, the introduced mutations significantly increased the transformation rates except ones in CYP11B1_Oa (Fig. 3c). And stains Ec02040110 and Ec02040111 processing mutated CYP11B1_Hs and CYP11B1_Bt achieved the highest transformation rate at 10.36 and 10.63 mg/L/h, respectively. Because CYP11B1_Hs mutant G25R/G46R/L52M (CYP11B1_Hs mutant1) achieved a little higher product/by-product ratio (0.35) than mutated CYP11B1_Bt (0.34), CYP11B1_Hs mutant1 was employed for further study.

For the reaction from 11-deoxycortisol to cortisol, Schiffer et al. [11] once reported that increasing substrate concentration led to enhancement on CYP11B1 activity. Here, the effect of substrate concentration on our biotransformation (from 17-OHP to 21-DF) was also characterized under different 17-OHP concentrations from 0.72 to 7.2 g/L (Additional file 1: Figure S5a). As shown in Additional file 1: Figure S5b, higher 17-OHP concentrations resulted in firstly increases and then decreases on the maximal conversion rate. And the highest transformation rate of 16.2 mg/L/h was achieved under the 17-OHP concentration at 2.4 g/L (Additional file 1: Figure S5b). This concentration was then applied in the following optimization process.

### Improve the product specificity via regulating the relative position between the catalytic active site heme and the substrate

**Fig. 4:**
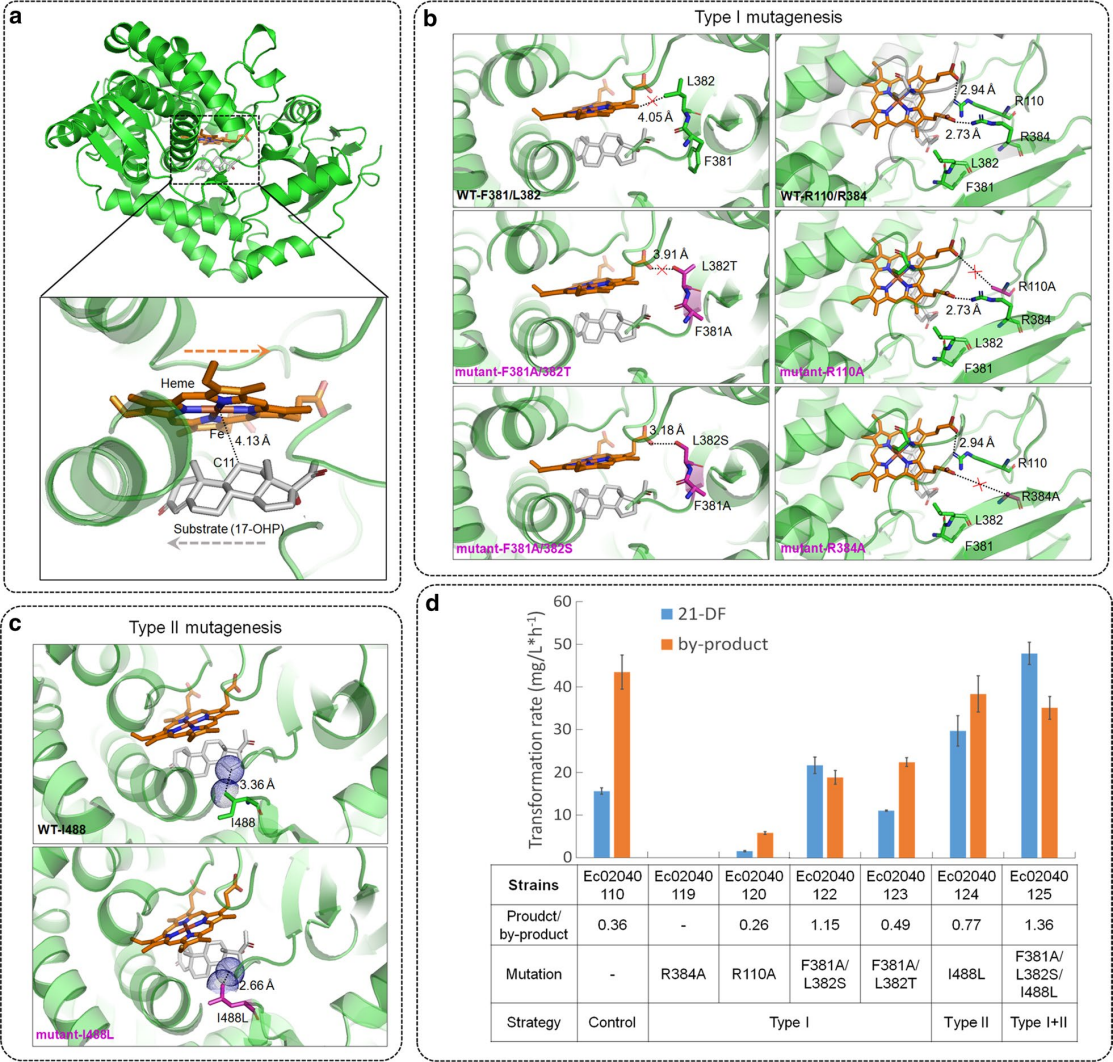
Improve the targeted product specificity by mutagenesis to adjust the relative position between the catalytic active site heme and the substrate. Heme in the catalytic active site of CYP11B1 was represented by *orange*, while the substrate 17-OHP was represented by *grey*. All the mutated residues were highlighted in *purple*. **a** The structural model of CYP11B1_Hs mutant1/17-OHP complex. The *orange* and *grey arrows* indicated the moving direction of the heme and the substrate, respectively. **b** The strategy for type I mutagenesis including F381A/L382S, F381A/L382T, R110A and R384A. **c** The strategy for type II mutagenesis as I488L. **d** The transformation rate of 21-DF and product/by-product ratio achieved by the designed mutagenesis

In this study, regardless of CYP11B1 species, there was always an unidentified by-product accumulated that was much more than our desired product 21-DF in the fermentation (Additional file 1: Figure S4). For instance, the product/by-product ratio of the control strain Ec02040110 with CYP11B1_Hs mutant1 was 0.35 (Fig. 3c). In order to improve the product specificity of CYP11B1, the structural model of CYP11B1_Hs mutant1 with 17-OHP was generated and analyzed. As shown in Fig. 4a, the Fe ion of heme in the catalytic active site of CYP11B1 adjoined C11 site of the substrate with a long distance of 4.13 Å, which implied the weak catalytic activity for hydroxylation on C11 site of 17-OHP. That would result in poor product conversion or selectivity of 11-hydroxylation, which was supported by the low product/by-products ratio achieved in our study (Fig. 3c). In order to improve the catalytic activity and selectivity of 11-hydroxylation, the relative position between the catalytic active site heme and the substrate was directionally regulated via designing and introducing two kinds of site-directed mutations (Type I and II) in the activity pocket of CYP11B1, according to the structures of enzyme-substrate complex (Fig. 4).

Type I mutagenesis was proposed to move the Fe ion of heme close to C11 site in the direction as the orange arrow indicated in Fig. 4a. The structural analysis revealed that R110 as well as R384 were essential for structural stability and catalysis function of heme (Fig. 4b). These two residues were also highly conserved among the nine selected CYP11B1s (Additional file 1: Figure S3b). When R110 and R384 were separately mutated into alanine within CYP11B1_Hs mutant1 in the control strain Ec02040110, the corresponding transformation rate for 21-DF decreased by 100 and 90.1%, respectively (Fig. 4d), indicating that weakening the interaction with the carboxyl of heme would decay the catalytic activity of CYP11B1 for generating the targeted product 21-DF. By contrast, if L382 was substituted by the residue with hydroxyl group in the side chain (i.e. serine or threonine), it would provide additional hydrogen bond interaction with the carboxyl of heme to draw the heme close to the C11 side of the substrate. Besides this mutagenesis site L382, mutation F381A was also introduced to enhance the effect of mutation L382 by the flexibility of alanine. As a result, compared with the data from the control strain, mutant F381A/L382S improved the product/by-product ratio and the transformation rate by 219 and by 39.1%, respectively (Fig. 4d). This outcome confirmed our design on type I mutagenesis which mainly improve the specificity of CYP11B1 to our targeted product. However, mutant F381A/L382T enhanced the product/by-product ratio only by 36.1%, and decreased the transformation rate for 21-DF by 28.8% (Fig. 4d). This unexpected result might due to that the methyl in the side chain of residue T382 forced the orientation of its adjacent hydroxyl group to be opposite to our desired direction. Under the circumstances, no additional interaction with heme was formed and this mutation probably damaged the functional structure of CYP11B1 (Fig. 4b). Thus, only mutant F381A/L382S could be applied latter.

Type II mutagenesis aimed to strengthen the substrate binding of CYP11B1 as well as to move the C11 site of substrate toward the Fe ion of heme in the direction as the grey arrow indicated in Fig. 4a. The complex structural model demonstrated that the side chain in residue I488 formed a hydrophobic force with the substrate (Fig. 4c). If I488 was substituted by a residue with larger hydrophobic group in the side chain (such as leucine), the distance between this residue and the substrate was shortened (e.g. from 3.36 to 2.66 Å for I488L), leading to an enhanced hydrophobic force which might improve substrate binding and slightly shift C11 site of 17-OHP towards the Fe ion of heme (Fig. 4c). As illustrated in Fig. 4d, compared with control strain Ec02040110, mutant I488L improved the transformation rate and the product/by-product ratio for 21-DF by 91.0 and by 114%, respectively. These data corroborated type II mutagenesis that improved the target product specificity as well as the catalytic activity of CYP11B1. Moreover, substrate binding to a P450 usually results in the binding spectra occurring from a shift from low-spin FeIII to high-spin FeIII upon binding of the substrate [73, 74]. Therefore, in order to confirm that mutant I488L improved the substrate binding of CYP11B1 besides the bioinformatical data, it is necessary to determine the binding spectra of CYP11B1 to 17-OHP as well as to compare the KS value of the mutant to the wild-type enzyme in future study.

Eventually, the mutations exhibiting positive effect (i.e. F381A/L382S from type I mutagenesis and I488L from type II mutagenesis) were all incorporated into the CYP11B1_Hs mutant1, obtaining CYP11B1_Hs mutant2. As a result, 1.96-fold enhancement on 21-DF transformation rate (to 47.9 mg/L/h) and 2.78-fold improvement on product/by-product ratio (to 1.36) were achieved in strain Ec02040125 harboring CYP11B1_Hs mutant2 (Fig. 4d). After 38-h biotransformation in shake-flask, the production of 21-DF reached to 1.42 g/L with a yield of 52.7%, which was the highest 21-DF production as known.

## Conclusions

In our study, *E. coli* MG1655(DE3) exhibited the highest up-taking rate of 17-OHP than other tested host cells. Then the biotransformation of 17-OHP to 21-DF was successfully achieved by establishing a whole-cell catalyst via incorporating heterologous CYP11B1 along with bovine Adx and AdR in *E. coli* MG1655(DE3). The effects of CYP11B1 with N-terminal mutagenesis from diverse sources and substrate concentration were investigated to stepwise increase 21-DF transformation rate by 343 and 56.4%, respectively. To be noted, in order to improve the catalytic activity as well as the product specificity by adjusting the relative position between the substrate 17-OHP and the catalytic active site heme in CYP11B1, two types of novel mutations were designed based on structural analysis of the enzyme-substrate complex. 1.96-fold enhancement on 21-DF transformation rate (to 47.9 mg/L/h) and 2.78-fold improvement on product/by-product ratio (to 1.36) were further achieved by the combination of two types of mutations. Eventually, the highest reported 21-DF production (1.42 g/L) along with a yield of 52.7% was obtained in strain Ec02040125 harboring CYP11B1_Hs mutant2 (G25R/G46R/L52M/F381A/L382S/I488L). This study offers a good example of whole cell biocatalyst system for efficient steroids biotransformation. It also provides important insights that can guide the audiences to regulate catalytic acidity as well as product specificity of other P450s.

## Additional file

**Additional file 1: Table S1.** Oligonucleotides used in this study. **Table S2.** Plasmids used in this study. **Table S3.** The Codon-optimized sequences of *CY11B1*, *Adx* and *AdR* involved in this study. **Figure S1.** Schematic representation of the constructing strategies for Adx_*4*–*108*_-CYP11B1-AdR co-expression plasmid (pETXST39–56). **Figure S2.** Schematic representation of the mutagenesis strategy applied in this study. **Figure S3.** Alignment of CYP11B1 s from nine species screened in this study. **Figure S4.** Conversion of 17-OHP to 21-DF by biocatalysts harboring different wild-type and mutated CYP11B1s from diversity species. **Figure S5.** Optimizing substrate concentration for higher biocatalyst efficiency. **Figure S6.** Biotransformation of 17-OHP to 21-DF by strains Ec02040126 and Ec02040110 during the time course.

## Abbreviations

21-DF: 21-deoxycortisol
17-OHP: 17α-hydroxyprdgesterone
CYP11B1: 11β-hydroxylase
AdR: adrenodoxin reductase
Adx: adrenodoxin
Ho: *Homo sapiens*
Bt: *Bos taurus*
Oa: *Ovis aries*
Sh: *Sarcophilus harrisii*
Pa: *Pteropus Alecto*
Cco: *Coprinopsis cinerea okayama*
Mo: *Magnaporthe oryzae*, Sa: *Sorex araneus*
