# Supplementary material for: Cell foundry with high product specificity and catalytic activity for 21-deoxycortisol biotransformation
Source: Microb Cell Fact. 2017 Jun 13;16:105. doi: 10.1186/s12934-017-0720-y (PMC5470312; doi:10.1186/s12934-017-0720-y)
Supplement: Supplementary file 1 — Additional file 1: Table S1. Oligonucleotides used in this study. Table S2. Plasmids used in this study. Table S3. The Codon-optimized sequences of CY11B1, Adx and AdR involved in this study. Figure S1. Schematic representation of the constructing strategies for Adx4–108-CYP11B1-AdR co-expression plasmid (pETXST39–56). Figure S2. Schematic representation of the mutagenesis strategy applied in this study. Figure S3. Alignment of CYP11B1 s from nine species screened in this study. Figure S4. Conversion of 17-OHP to 21-DF by biocatalysts harboring different wild-type and mutated CYP11B1s from diversity species. Figure S5. Optimizing substrate concentration for higher biocatalyst efficiency. Figure S6. Biotransformation of 17-OHP to 21-DF by strains Ec02040126 and Ec02040110 during the time course. [file 12934_2017_720_MOESM1_ESM.docx]

**Supporting information**

**Cell foundry with high product specificity and catalytic activity for 21-deoxycortisol biotransformation**

Shuting Xiong^a,b＃^, Ying Wang^a,b＃^, Mingdong Yao ^a,b^, Hong Liu^a,b^, Xiao Zhou ^a,b^, Wenhai Xiao^a,b*^,Yingjin Yuan^a,b^

^a^ Key Laboratory of Systems Bioengineering (Ministry of Education), Tianjin University, Tianjin, 300072, PR China.

^b^ SynBio Research Platform, Collaborative Innovation Center of Chemical Science and Engineering (Tianjin), School of Chemical Engineering and Technology, Tianjin University, Tianjin, 300072, PR China.

*Corresponding author:

Ying Wang (Email: wenhai.xiao@tju.edu.cn, Tel: 86-22-60973987, Postal address: No. 92, Weijin Road, Nankai District, Tianjin, 300072, PR China)

# These authors contributed equally to this work

**Table S1.** Oligonucleotides used in this study

| **Oligo name** | **Sequence (5’- 3’)** |
| --- | --- |
| **For introducing mutations G46R and L52M in pUC57*-CYP11B1*_Hs_G25R_** | |
| F_ Hs | GGAATTCCATATGGCGCTGCGTGCGAAAGC |
| R_ Hs | GGACTAGTTAGTTGATCGCACG |
| OE-1-Hs | CTTGCTCACGCCAGATTTGCAGCATACGCAGCCAACGGTTACGCGGACGACGCGGCATCGCTTC |
| OE-2-Hs | GAAGCGATGCCGCGTCGTCCGCGTAACCGTTGGCTGCGTATGCTGCAAATCTGGCGTGAGCAAG |
| **For introducing mutation G46R in pUC57*-CYP11B1*_Rn_G25R_** | |
| F_ Rn | GGAATTCCATATGGCGCTGTGGGCGAAAGC |
| R_ Rn | GGACTAGTTATTGGATCGCACG |
| OE-1-Rn | GCATACGCATCCACTTGTTACGCGGGCAACGCGGCATCGCC |
| OE-2-Rn | GGCGATGCCGCGTTGCCCGCGTAACAAGTGGATGCGTATGC |
| **For introducing mutations G46R and V52M in pUC57*-CYP11B1*_Oa_G25R_** | |
| F_ Oa | GGAATTCCATATGGCGCTGTGGGCGAAAGC |
| R_ Oa | GGACTAGTTATTGGATCGCGCC |
| OE-1-Oa | GCTCTTTCCAGATTTGCAGACGCATCCACTTGTTACGCGGGCAACGCGGCATCGC |
| OE-2-Oa | GCGATGCCGCGTTGCCCGCGTAACAAGTGGATGCGTCTGCAAATCTGGAAAGAGC |
| **For introducing mutation S55R in pUC57*-CYP11B1*_Sh_T26R_** | |
| F_ Sh | GGAATTCCATATGAAGCAGGTGACCGAGCTG |
| R_ Sh | GGACTAGTTAGTTCAGTTCACGC |
| OE-1-Sh | GCATCTTGGTCCACGGGTTACGTTGGCTCTGCGGGATCGCTTC |
| OE-2-Sh | GAAGCGATCCCGCAGAGCCAACGTAACCCGTGGACCAAGATGC |
| **For introducing mutations G46R and A52M in pUC57-*CYP11B1*_Hs_G25R_** | |
| F_ Pa | GGAATTCCATATGGCGCTGCGTGCGCGTGCG |
| R_ Pa | GGACTAGTTACGGGCTGCTGGTTTG |
| OE-1-Pa | GCTCACGCCACACTTGCAGCATACGAACCCATTTGTTACGCGGGCAACGCGGGATCGCC |
| OE-2-Pa | GGCGATCCCGCGTTGCCCGCGTAACAAATGGGTTCGTATGCTGCAAGTGTGGCGTGAGC |
| **For introducing mutations W38R and N44M in** **pUC57*-CYP11B1*_Cco_Y20R_** | |
| F_ Cco | GGAATTCCATATGGCGAGCAACGCGCTGCTG |
| R_ Cco | GGACTAGTTATTGCTGACGCGG |
| OE-1-Cco | GGTGATAGCCAATCCAGATCATGCTAATCGCCGCGTAACGCGGACCCGGGATCGCGCTCAG |
| OE-2-Cco | CTGAGCGCGATCCCGGGTCCGCGTTACGCGGCGATTAGCATGATCTGGATTGGCTATCACC |
| **For introducing mutations S50R and D56M in pUC57-*CYP11B1*_Mo_A30R_** | |
| F_ Mo | GGAATTCCATATGCTGAACATGAGCTTCAGC |
| R_ Mo | GTAAGACCCCGGTTTAACTAGTCC |
| OE-1-Mo | GGCTACGAATCATCCAGTACATGGTCCAACCCGCCAGACGCGGACCATCAATATGTTTC |
| OE-2-Mo | GAAACATATTGATGGTCCGCGTCTGGCGGGTTGGACCATGTACTGGATGATTCGTAGCC |
| **For introducing mutations V41R and L47M in pUC57*-CYP11B1*_Sa_G25R_** | |
| F_ Sa | GGAATTCCATATGGCGTTTCGTGCGCAGGTG |
| R_ Sa | GGACTAGTTAGTTCAGTTCACGC |
| OE-1-Sa | CAAAGCCTTTACCCCACAGCATCTTCATACCCGGGCTACGCGGATACTGCGGCACCGCCGC |
| OE-2-Sa | GCGGCGGTGCCGCAGTATCCGCGTAGCCCGGGTATGAAGATGCTGTGGGGTAAAGGCTTTG |
| **For introducing mutation in pETXST48** | |
| F_ Adx | GCTCTAGAAATAATTTTGTTTAACTTTAAGAAGGAGATATACATATGCGTCTGCTGCGTGTTG |
| R_ AdR | CGGATCCGCGACCCATTTGCTGTCCACCAGTCATGATGGCACTAGTTAGTGGCCCAGCAGAC |
| **For introducing mutation I488L in pETXST48** | |
| OE-1-I488L | GAACATGCTCGGACGCAGAAGGAAGCTGTAAACCATCTTGATATCTTC |
| OE-2-I488L | GAAGATATCAAGATGGTTTACAGCTTCCTTCTGCGTCCGAGCATGTTC |
| **For introducing mutation R384A in pETXST48** | |
| OE-1-R384A | CAGGTCGCTGCTCGCCACAGCTTCCAGGAACAGACCCACCG |
| OE-2-R384A | CGGTGGGTCTGTTCCTGGAAGCTGTGGCGAGCAGCGACCTG |
| **For introducing mutation R110A in pETXST48** | |
| OE-1-R110A | CACGGCTCCAGGCTCATAGCGTGCGGGTGCAGGCTATC |
| OE-2-R110A | GATAGCCTGCACCCGCACGCTATGAGCCTGGAGCCGTG |
| **For introducing mutations FL381AS in pETXST48** | |
| OE-1-FL381AS | CTGCTCGCCACACGTTCAGTACTCAGACCCACCGGATACAGAC |
| OE-2-FL381AS | GTCTGTATCCGGTGGGTCTGGCTAGTGAACGTGTGGCGAGCAG |
| **For introducing mutations FL381AT in pETXST48** | |
| OE-1-FL381AT | CTGCTCGCCACACGTTCAGTAGCCAGACCCACCGGATACAGAC |
| OE-2-FL381AT | GTCTGTATCCGGTGGGTCTGGCTACTGAACGTGTGGCGAGCAG |

**Table S2.** Plasmids used in this study

| **plasmid** | **Description** | **Source** |
| --- | --- | --- |
| pUC57-Simple | Blunt Cloning vector, resistant to ampicillin and kanamycin | GenScript |
| pGro7 | Chaperone vector for expression of GroES & GroEL to assist protein folding, induced by L-arabinose, resistant to chloromycetin | TaKaRa |
| pET21a-YX | T7 expression vector, containing EcoRI, XbaI, SpeI and PstI sites for BioBrick^TM^ assembly strategy, resistant to ampicillin | [1] |
| pUC57-*Adx_4-108_* | *Adx_4-108_* from *Bos taurus* was codon optimized, synthesized with 5’-NdeI site and 3’-SpeI site and cloned into pUC57-Simple | This study |
| pUC57-*AdR* | *AdR* from *Bos taurus* was codon optimized, synthesized with 5’ add NdeI while 3’ add SpeI and cloned into pUC57-Simple | This study |
| pUC57-*CYP11B1*_Hs | *CYP11B1* from *Homo sapiens* (*CYP11B1*_Hs) was codon optimized, synthesized with 5’-NdeI site and 3’-SpeI site and cloned into pUC57-Simple | This study |
| pUC57-*CYP11B1_Bt* | *CYP11B1* from *Bos taurus* (*CYP11B1*_Bt) was codon optimized, synthesized with 5’-NdeI site and 3’-SpeI site and cloned into pUC57-Simple | This study |
| pUC57-*CYP11B1*_Rn | *CYP11B1* from *Rattus norvegicus* (*CYP11B1*_Rn) was codon optimized, synthesized with 5’-NdeI site and 3’-SpeI site and cloned into pUC57-Simple | This study |
| pUC57*-CYP11B1_Oa* | *CYP11B1* from *Ovis aries* (*CYP11B1*_Oa) was codon optimized, synthesized with 5’-NdeI site and 3’-SpeI site and cloned into pUC57-Simple | This study |
| pUC57-*CYP11B1_Sh* | *CYP11B1* from *Sarcophilus harrisii* (*CYP11B1*_Sh) was codon optimized, synthesized with 5’-NdeI site and 3’-SpeI site and cloned into pUC57-Simple | This study |
| pUC57*-CYP11B1_Pa* | *CYP11B1* from *Pteropus alecto* (*CYP11B1*_Pa) was codon optimized, synthesized with 5’-NdeI site and 3’-SpeI site and cloned into pUC57-Simple | This study |
| pUC57-*CYP11B1_Cco* | *CYP11B1* from *Coprinopsis cinerea okayama* (*CYP11B1*_Cco) was codon optimized, synthesized with 5’-NdeI site and 3’-SpeI site and cloned into pUC57-Simple | This study |
| pUC57-*CYP11B1_Mo* | *CYP11B1* from *Magnaporthe oryzae* (*CYP11B1*_Mo) was codon optimized, synthesized with 5’-NdeI site and 3’-SpeI site and cloned into pUC57-Simple | This study |
| pUC57-*CYP11B1_Sa* | *CYP11B1* from *Sorex araneus* (*CYP11B1*_Sa) was codon optimized, synthesized with 5’-NdeI site and 3’-SpeI site and cloned into pUC57-Simple | This study |
| pUC57*-CYP11B1*_Hs_G25R_ | *CYP11B1* from *Homo sapiens* (*CYP11B1*_Hs_G25R_) with a G25R mutation was codon optimized, synthesized with 5’-NdeI site and 3’-SpeI site and cloned into pUC57-Simple | This study |
| pUC57-*CYP11B1*_Bt_G25R_ | *CYP11B1* from *Bos taurus* (*CYP11B1*_Bt_G25R_) with a G25R mutation was codon optimized, synthesized with 5’-NdeI site and 3’-SpeI site and cloned into pUC57-Simple | This study |
| pUC57-*CYP11B1*_Rn_G25R_ | *CYP11B1* from *Rattus norvegicus* (*CYP11B1*_Rn_G25R_) with a G25R mutation was codon optimized, synthesized with 5’-NdeI site and 3’-SpeI site and cloned into pUC57-Simple | This study |
| pUC57-*CYP11B1*_Oa_G25R_ | *CYP11B1* from *Ovis aries* (*CYP11B1*_Oa_G25R_) with a G25R mutation was codon optimized, synthesized with 5’-NdeI site and 3’-SpeI site and cloned into pUC57-Simple | This study |
| pUC57-*CYP11B1*_Sh_T26R_ | *CYP11B1* from *Sarcophilus harrisii* (*CYP11B1*_Sh_T26R_) with a T26R mutation was codon optimized, synthesized with 5’-NdeI site and 3’-SpeI site and cloned into pUC57-Simple | This study |
| pUC57-*CYP11B1*_Pa_G25R_ | *CYP11B1* from *Pteropus alecto* (*CYP11B1*_Pa_G25R_) with a G25R mutation was codon optimized, synthesized with 5’-NdeI site and 3’-SpeI site and cloned into pUC57-Simple | This study |
| pUC57-*CYP11B1*_Cco_Y20R_ | *CYP11B1* from *Coprinopsis cinerea okayama* (*CYP11B1*_Cco_Y20R_) with a Y20R mutation was codon optimized, synthesized with 5’-NdeI site and 3’-SpeI site and cloned into pUC57-Simple | This study |
| pUC57-*CYP11B1*_Mo_A30R_ | *CYP11B1* from *Magnaporthe oryzae* (*CYP11B1*_Mo_A30R_) with a A30R mutation was codon optimized, synthesized with 5’-NdeI site and 3’-SpeI site and cloned into pUC57-Simple | This study |
| pUC57*-CYP11B1*_Sa_A30R_ | *CYP11B1* from *Sorex araneus* (*CYP11B1*_Sa_A30R_) with a A30R mutation was codon optimized, synthesized with 5’-NdeI site and 3’-SpeI site and cloned into pUC57-Simple | This study |
| pETXST01 | pET21a-*Adx_4-108_*, *Adx_4-108_* from *Bos taurus* was digested from pUC57-*Adx_4-108_* by NdeI/SpeI and inserted into the same sites of the pET21a-YX | This study |
| pETXST02 | pET21a-*AdR*, *AdR* from *Bos taurus* was digested from pUC57-*AdR* by NdeI*/* SpeI and inserted into the same sites of the pET21a-YX | This study |
| pETXST03 | pET21a-*CYP11B1*_Hs, *CYP11B1*_Hs was digested from pUC57-*CYP11B1*_Hs by NdeI/ SpeI and inserted into the same sites of the pET21a-YX | This study |
| pETXST04 | pET21a-*CYP11B1*_Bt, *CYP11B1*_Bt was digested from pUC57-*CYP11B1-Bt* by NdeI/ SpeI and inserted into the same sites of the pET21a-YX | This study |
| pETXST05 | pET21a-*CYP11B1*_Rn, *CYP11B1*_Rn was digested from pUC57-*CYP11B1*_Rn by NdeI*/* SpeI and inserted into the same sites of the pET21a-YX | This study |
| pETXST06 | pET21a-*CYP11B1_Oa*, *CYP11B1_Oa* was digested from pUC57*-CYP11B1_Oa* by NdeI*/* SpeI and inserted into the same sites of the pET21a-YX | This study |
| pETXST07 | pET21a-*CYP11B1_Sh*, *CYP11B1_Sh* was digested from pUC57-*CYP11B1_Sh* by NdeI*/* SpeI and inserted into the same sites of the pET21a-YX | This study |
| pETXST08 | pET21a-*CYP11B1_Pa*, *CYP11B1_Pa* was digested from pUC57*-CYP11B1_Pa* by NdeI*/* SpeI and inserted into the same sites of the pET21a-YX | This study |
| pETXST09 | pET21a-*CYP11B1_Cco*, *CYP11B1_Cco* was digested from pUC57-*CYP11B1_Cco* by NdeI*/* SpeI and inserted into the same sites of the pET21a-YX | This study |
| pETXST10 | pET21a-*CYP11B1_Mo*, *CYP11B1_Mo* was digested from pUC57-*CYP11B1_Mo* by NdeI*/* SpeI and inserted into the same sites of the pET21a-YX | This study |
| pETXST11 | pET21a-*CYP11B1_Sa*, *CYP11B1_Sa* was digested from pUC57-*CYP11B1_Sa* by NdeI*/* SpeI and inserted into the same sites of the pET21a-YX | This study |
| pETXST12 | pET21a-*CYP11B1*_Hs_G25R/G46R/L52M_, mutations G46R and L52M within gene *CYP11B1* from plasmid pUC57*-CYP11B1*_Hs_G25R_ | This study |
| pETXST13 | pET21a-*CYP11B1*_Bt_G25R_, *CYP11B1*_Bt_G25R_ was digested from pUC57-*CYP11B1*_Bt_G25R_ and inserted into NdeI*/*SpeI site of the pET21a-YX | This study |
| pETXST14 | pET21a-*CYP11B1*_Rn_G25R/G46R_, mutation G46R within gene *CYP11B1* from plasmid pUC57-*CYP11B1*_Rn_G25R_ | This study |
| pETXST15 | pET21a-*CYP11B1*_Oa_G25R/G46R/V52M_, mutations G46R and V52M within gene *CYP11B1* from plasmid pUC57-*CYP11B1*_Oa_G25R_ | This study |
| pETXST16 | pET21a-*CYP11B1*_Sh_T26R/S55R_, mutations T26R and S55R within gene *CYP11B1* from plasmid pUC57-*CYP11B1*_Sh_T26R_ | This study |
| pETXST17 | pET21a-*CYP11B1*_Pa_G25R/G46R/A52M_, mutations G46R and L52M within gene *CYP11B1* from plasmid pUC57-*CYP11B1*_Pa_G25R_ | This study |
| pETXST18 | pET21a-*CYP11B1*_Cco_Y20R/W38R/N44M_, mutations W38R and N44M within gene *CYP11B1* from plasmid pUC57-*CYP11B1*_Cco_Y20R_ | This study |
| pETXST19 | pET21a-*CYP11B1*_Mo_A30R/S50R/D56M_, mutations S50R and D56M within gene *CYP11B1* from plasmid pUC57-*CYP11B1*_Mo_A30R_ | This study |
| pETXST20 | pET21a- *CYP11B1*_Sa_A30R/V41R/L47M_, mutations V41R and L47M within gene *CYP11B1* from plasmid pUC57*-CYP11B1*_Sa_G25R_ | This study |
| pETXST21 | pET21a-*Adx_4-108_-CYP11B1*_Hs, *CYP11B1*_Hs was cut by XbaI/BamHI from pETXST03 and inserted into the SpeI/BamHI sites of pETXST01 | This study |
| pETXST22 | pET21a-*Adx_4-108_-CYP11B1*_Bt, *CYP11B1*_Bt was cut by XbaI/BamHI from pETXST04 and inserted into the SpeI/BamHI sites of pETXST01 | This study |
| pETXST23 | pET21a-*Adx_4-108_-CYP11B1*_Rn, *CYP11B1*_Rn was cut by XbaI/BamHI from pETXST05 and inserted into the SpeI/BamHI sites of pETXST01 | This study |
| pETXST24 | pET21a-*Adx_4-108_-CYP11B1_Oa*, *CYP11B1_Oa* was cut by XbaI/BamHI from pETXST06 and inserted into the SpeI/BamHI sites of pETXST01 | This study |
| pETXST25 | pET21a-*Adx_4-108_*-*CYP11B1_Sh*, *CYP11B1_Sh* was cut by XbaI/BamHI from pETXST07 and inserted into the SpeI/BamHI sites of pETXST01 | This study |
| pETXST26 | pET21a-*Adx_4-108_-CYP11B1_Pa*, *CYP11B1_Pa* was cut by XbaI/BamHI from pETXST08 and inserted into the SpeI/BamHI sites of pETXST01 | This study |
| pETXST27 | pET21a-*Adx_4-108_-CYP11B1_Cco*, *CYP11B1_Cco* was cut by XbaI/BamHI from pETXST09 and inserted into the SpeI/BamHI sites of pETXST01 | This study |
| pETXST28 | pET21a-*Adx_4-108_-CYP11B1_Mo*, *CYP11B1_Mo* was cut by XbaI/BamHI from pETXST10 and inserted into the SpeI/BamHI sites of pETXST01 | This study |
| pETXST29 | pET21a-*Adx_4-108_-CYP11B1_Sa*, *CYP11B1_Sa* was cut by XbaI/BamHI from pETXST11 and inserted into the SpeI/BamHI sites of pETXST01 | This study |
| pETXST30 | pET21a-*Adx_4-108_-CYP11B1*_Hs_G25R/G46R/L52M_, *CYP11B1*_Hs_G25R/G46R/L52M_ was cut by XbaI/BamHI from pETXST12 and inserted into the SpeI/BamHI sites of pETXST01 | This study |
| pETXST31 | pET21a-*Adx_4-108_-CYP11B1*_Bt_G25R_, *CYP11B1*_Bt_G25R_ was cut by XbaI/BamHI from pETXST13 and inserted into the SpeI/BamHI sites of pETXST01 | This study |
| pETXST32 | pET21a-*Adx_4-108_-CYP11B1*_Rn_G25R/G46R_, *CYP11B1*_Rn_G25R/G46R_ as cut by XbaI/BamHI from pETXST14 and inserted into the SpeI/BamHI sites of pETXST01 | This study |
| pETXST33 | pET21a-*Adx_4-108_-CYP11B1*_Oa_G25R/G46R/V52M_, *CYP11B1*_Oa_G25R/G46R/V52M_ was cut by XbaI/BamHI from pETXST15 and inserted into the SpeI/BamHI sites of pETXST01 | This study |
| pETXST34 | pET21a-*Adx_4-108_-CYP11B1*_Sh_T26R/S55R_, *CYP11B1*_Sh_T26R/S55R_ was cut by XbaI/BamHI from pETXST16 and inserted into the SpeI/BamHI sites of pETXST01 | This study |
| pETXST35 | pET21a-*Adx_4-108_-CYP11B1*_Pa_G25R/G46R/A52M_, *CYP11B1*_Pa_G25R/G46R/A52M_ was cut by XbaI/BamHI from pETXST17 and inserted into the SpeI/BamHI sites of pETXST01 | This study |
| pETXST36 | pET21a-*Adx_4-108_-CYP11B1*_Cco_Y20R/W38R/N44M_, *CYP11B1*_Cco_Y20R/W38R/N44M_ was cut by XbaI/BamHI from pETXST18 and inserted into the SpeI/BamHI sites of pETXST01 | This study |
| pETXST37 | pET21a-*Adx_4-108_*-*CYP11B1*_Mo_A30R/S50R/D56M_, *CYP11B1*_Mo_A30R/S50R/D56M_ was cut by XbaI/BamHI from pETXST19 and inserted into the SpeI/BamHI sites of pETXST01 | This study |
| pETXST38 | pET21a-*Adx_4-108_-CYP11B1*_Sa_A30R/V41R/L47M_, *CYP11B1*_Sa_A30R/V41R/L47M_ was cut by XbaI/BamHI from pETXST20 and inserted into the SpeI/BamHI sites of pETXST01 | This study |
| pETXST39 | pET21a-*Adx_4-108_-CYP11B1_*Hs-*AdR*, AdR was cut by XbaI/BamHI from pETXST02 and inserted into the SpeI/BamHI sites of pETXST21 | This study |
| pETXST40 | pET21a-*Adx_4-108_-CYP11B1*_Bt*-AdR*, AdR was cut by XbaI/BamHI from pETXST02 and inserted into the SpeI/BamHI sites of pETXST22 | This study |
| pETXST41 | pET21a-*Adx_4-108_-CYP11B1*_Rn*-AdR*, AdR was cut by XbaI/BamHI from pETXST02and inserted into the SpeI/BamHI sites of pETXST23 | This study |
| pETXST42 | pET21a-*Adx_4-108_-CYP11B1_Oa-AdR*, AdR was cut by XbaI/BamHI from pETXST02 and inserted into the SpeI/BamHI sites of pETXST24 | This study |
| pETXST43 | pET21a-*Adx_4-108_-CYP11B1_Sh-AdR*, AdR was cut by XbaI/BamHI from pETXST02 and inserted into the SpeI/BamHI sites of pETXST25 | This study |
| pETXST44 | pET21a-*Adx_4-108_ CYP11B1_Pa-AdR*, AdR was cut by XbaI/BamHI from pETXST02 and inserted into the SpeI/BamHI sites of pETXST26 | This study |
| pETXST45 | pET21a-*Adx_4-108_-CYP11B1_Cco-AdR*, AdR was cut by XbaI/BamHI from pETXST02 and inserted into the SpeI/BamHI sites of pETXST27 | This study |
| pETXST46 | pET21a-*Adx_4-108_-CYP11B1_Mo-AdR*, AdR was cut by XbaI/BamHI from pETXST02 and inserted into the SpeI/BamHI sites of pETXST28 | This study |
| pETXST47 | pET21a-*Adx_4-108_-CYP11B1_Sa-AdR*, AdR was cut by XbaI/BamHI from pETXST02 and inserted into the SpeI/BamHI sites of pETXST29 | This study |
| pETXST48 | pET21a-*Adx_4-108_-CYP11B1_*Hs_G25R/G46R/L52M_-*AdR*, AdR was cut by XbaI/BamHI from pETXST02 and inserted into the SpeI/BamHI sites of pETXST30 | This study |
| pETXST49 | pET21a-*Adx_4-108_-CYP11B1*_Bt_G25R_*-AdR*, AdR was cut by XbaI/BamHI from pETXST02 and inserted into the SpeI/BamHI sites of pETXST31 | This study |
| pETXST50 | pET21a-*Adx_4-108_-CYP11B1*_Rn_G25R/G46R_*-AdR*, AdR was cut by XbaI/BamHI from pETXST02 and inserted into the SpeI/BamHI sites of pETXST32 | This study |
| pETXST51 | pET21a-*Adx_4-108_-CYP11B1*_Oa_G25R/G46R/V52M_*-AdR*, AdR was cut by XbaI/BamHI from pETXST02 and inserted into the SpeI/BamHI sites of pETXST33 | This study |
| pETXST52 | pET21a-*Adx_4-108_-CYP11B1*_Sh_T26R/S55R_*-AdR*, AdR was cut by XbaI/BamHI from pETXST02 and inserted into the SpeI/BamHI sites of pETXST34 | This study |
| pETXST53 | pET21a-*Adx_4-108_-CYP11B1*_Pa_G25R/G46R/A52M_*-AdR*, AdR was cut by XbaI/BamHI from pETXST02 and inserted into the SpeI/BamHI sites of pETXST35 | This study |
| pETXST54 | pET21a-*Adx_4-108_-CYP11B1*_Cco_Y20R/W38R/N44M_*-AdR*, AdR was cut by XbaI/BamHI from pETXST02 and inserted into the SpeI/BamHI sites of pETXST36 | This study |
| pETXST55 | pET21a-*Adx_4-108_-CYP11B1*_Mo_A30R/S50R/D56M_*-AdR*, AdR was cut by XbaI/BamHI from pETXST02 and inserted into the SpeI/BamHI sites of pETXST37 | This study |
| pETXST56 | pET21a-*Adx_4-108_-CYP11B1*_Sa_A30R/V41R/L47M_*-AdR*, AdR was cut by XbaI/BamHI from pETXST02 and inserted into the SpeI/BamHI sites of pETXST38 | This study |
| pETXST57 | pET21a-*Adx_4-108_-CYP11B1_*Hs_G25R/G46R/L52M/R384A_-*AdR*, mutation R384A within gene *CYP11B1* from plasmid pETXST39 | This study |
| pETXST58 | pET21a-*Adx_4-108_-CYP11B1_*Hs_G25R/G46R/L52M/R110A_-*AdR*, mutation R110A within gene *CYP11B1* from plasmid p pETXST39 | This study |
| pETXST59 | pET21a-*Adx_4-108_-CYP11B1_*Hs_G25R/G46R/L52M/F381A/L382S_-*AdR*, mutations FL381AS within gene *CYP11B1* from plasmid pETXST39 | This study |
| pETXST60 | pET21a-*Adx_4-108_-CYP11B1_*Hs_G25R/G46R/L52M/F381A/L382T_-*AdR*, mutations FL381AT within gene *CYP11B1* from plasmid pETXST39 | This study |
| pETXST61 | pET21a-*Adx_4-108_-CYP11B1_*Hs_G25R/G46R/L52M/F381A/L382S/I488L_-*AdR*, mutation I488L within gene *CYP11B1* from plasmid pETXST39 | This study |
| p pETXST62 | pET21a-*Adx_4-108_-CYP11B1_*Hs_G25R/G46R/L52M/F381A/L382S/I488L_-*AdR*, mutations FL381AS and I488L within gene *CYP11B1* from plasmid pETXST39 | This study |

**Reference:**

**1. Cao YX, Xiao WH, Liu D, Zhang JL, Ding MZ, Yuan YJ. Biosynthesis of odd-chain fatty alcohols in Escherichia coli. *Metabolic Engineering* 2015, 29:113.**

**Table S3.** The Codon-optimized sequences of *CY11B1*, *Adx* and *AdR* involved in this study

| **Protein** | **Encoding sequences** |
| --- | --- |
| CYP11B1 from *Homo sapiens*  (CYP11B1_Hs) | atggcgctgcgtgcgaaagcggaagtgtgcatggcggttccgtggctgagcctgcaacgtgcgcaagcgctgggtacccgtgcggcgcgtgtgccgcgtaccgttctgccgtttgaagcgatgccgcgtcgtccgggtaaccgttggctgcgtctgctgcaaatctggcgtgagcaaggctacgaagacctgcacctggaagtgcaccagaccttccaagaactgggtccgatttttcgttatgatctgggtggcgcgggcatggtgtgcgttatgctgccggaggacgtggaaaagctgcaacaagttgatagcctgcacccgcaccgtatgagcctggagccgtgggtggcgtaccgtcagcaccgtggtcacaagtgcggcgtttttctgctgaacggtccggagtggcgtttcaaccgtctgcgtctgaacccggaagtgctgagcccgaacgcggttcaacgttttctgccgatggtggacgcggttgcgcgtgatttcagccaggcgctgaagaaaaaggtgctgcaaaacgcgcgtggtagcctgaccctggacgttcagccgagcatctttcactataccattgaggcgagcaacctggcgctgttcggcgaacgtctgggtctggttggccatagcccgagcagcgcgagcctgaacttcctgcacgcgctggaagtgatgtttaagagcaccgttcagctgatgttcatgccgcgtagcctgagccgttggaccagcccgaaagtgtggaaggagcacttcgaagcgtgggactgcatctttcaatacggtgataactgcatccagaaaatttatcaagagctggcgtttagccgtccgcagcaatacaccagcatcgttgcggagctgctgctgaacgcggaactgagcccggacgcgattaaggcgaacagcatggaactgaccgcgggcagcgtggataccaccgttttcccgctgctgatgaccctgtttgagctggcgcgtaacccgaacgtgcagcaagcgctgcgtcaggaaagcctggctgcggcggcgagcattagcgagcacccgcaaaaagcgaccaccgaactgccgctgctgcgtgcggcgctgaaggaaaccctgcgtctgtatccggtgggtctgttcctggaacgtgtggcgagcagcgacctggttctgcaaaactaccacatcccggcgggtaccctggtgcgtgtttttctgtatagcctgggtcgtaacccggcgctgtttccgcgtccggagcgttacaacccgcagcgttggctggatattcgtggtagcggccgtaacttttatcacgtgccgttcggttttggcatgcgtcagtgcctgggccgtcgtctggcggaggcggaaatgctgctgctgctgcaccacgtgctgaaacacctgcaagttgaaaccctgacccaagaagatatcaagatggtttacagcttcattctgcgtccgagcatgttcccgctgctgacctttcgtgcgatcaactaa |
| CYP11B1 from *Bos Taurus*  (CYP 11B1_Bt) | atggcgctgcgtgttaccgcggacgtttggctggcgcgtccgtggcaatgcctgcatcgtacccgtgcgctgggtaccaccgcgaaggtggcgccgaagaccctgaaaccgttcgaggcgatcccgcaatacagccgtaacaagtggctgaaaatgatccagattctgcgtgagcagggtcaagaaaacctgcacctggagatgcaccaggcgttccaagaactgggcccgatctttcgtcacagcgcgggtggcgcgcaaattgtgagcgttatgctgccggaggacgcggaaaagctgcaccaggttgaaagcatcctgccgcaccgtatgccgctggagccgtgggtggcgcaccgtgaactgcgtggtctgcgtcgtggcgtttttctgctgaacggcgcggattggcgtttcaaccgtctgcaactgaacccgaacatgctgagcccgaaagcgattcagagcttcgtgccgtttgttgacgtggttgcgcgtgattttgtggagaacctgaagaaacgtatgctggaaaacgttcacggtagcatgagcatcaacatccaaagcaacatgttcaactacacgatggaggcgagccacttcgtgattagcggtgaacgtctgggtctgaccggtcacgatctgaagccggagagcgttaccttcacccacgcgctgcacagcatgtttaagagcaccacccagctgatgttcctgccgaaaagcctgacccgttggaccagcacccgtgtgtggaaagagcacttcgacagctgggatatcattagcgaatacgtgaccaagtgcatcaaaaacgtttatcgtgagctggcggaaggtcgtcagcaaagctggagcgttattagcgagatggtggcgcagagcaccctgagcatggacgcgatccacgcgaacagcatggaactgattgcgggcagcgtggataccaccgcgatcagcctggttatgaccctgtttgaactggcgcgtaacccggacgtgcagcaagcgctgcgtcaagagagcctggcggcggaagcgagcattgttgcgaacccgcagaaggcgatgagcgatctgccgctgctgcgtgcggcgctgaaagaaaccctgcgtctgtacccggtgggtagcttcgttgaacgtatcgtgcacagcgacctggttctgcaaaactatcacgtgccggcgggtacctttgttatcatttacctgtatagcatgggccgtaacccggcggtgtttccgcgtccggagcgttacatgccgcagcgttggctggaacgcaagcgtagctttcaacacctggcgttcggttttggcatgcgtcagtgcctgggccgtcgtctggcggaagtggaaatgctgctgctgctgcaccacatgctgaaaaccttccaggtggaaaccctgcgtcaggaagatatgcaaatggttttccgttttctgctgatgccgagcagcagcccgttcctgacctttcgtccggttagctaa |
| CYP11B1 from *Rattus norvegicus*  (CYP 11B1_Rn) | atggcgctgtgggcgaaagcgcgtgtgcgtatggcgggtccgtggctgagcctgcatgaagcgcgtctgctgggtacccgtggtgcggcggcgccgaaagcggttctgccgtttgaggcgatgccgcgttgcccgggtaacaagtggatgcgtatgctgcaaatctggaaagagcaaagcagcgaaaacatgcacctggacatgcaccagaccttccaagaactgggcccgatttttcgttacgatgtgggtggccgtcacatggtgttcgttatgctgccggaggacgttgaacgtctgcaacaagcggatagccaccacccgcagcgtatgatcctggagccgtggctggcgtatcgtcaagcgcgtggtcacaagtgcggcgtgtttctgctgaacggtccgcagtggcgtctggaccgtctgcgtctgaacccggatgttctgagcctgccggcgctgcaaaagtacaccccgctggttgacggcgttgcgcgtgatttcagccagaccctgaaagcgcgtgtgctgcaaaacgcgcgtggtagcctgaccctgggtcaccgtgcgcagctgtttcgttacaccatcgaagcgagcaccctggttctgtatggcgagcgtctgggcctgctgacccagcaaccgaacccggacagcctgaacttcattcacgcgctggaagcgatgctgaagagcaccgtgcagctgatgtttgttccgcgtcgtctgagccgttggatgagcaccaacatgtggcgtgagcacttcgaagcgtgggattacatctttcaatatgcgaaccgtgcgatccagcgtatttaccaagaactggcgctgggtcacccgtggcactatagcggcattgtggcggagctgctgatgcgtgcggacatgaccctggataccatcaaagcgaacaccattgacctgaccgcgggtagcgttgataccaccgcgttcccgctgctgatgaccctgtttgaactggcgcgtaacccggaagtgcagcaagcggttcgtcaggagagcctggtggcggaagcgcgtatcagcgagaacccgcaacgtgcgattaccgaactgccgctgctgcgtgcggcgctgaaggaaaccctgcgtctgtatccggttggcatcaccctggagcgtgaagtgagcagcgacctggttctgcaaaactaccacattccggcgggtaccctggtgaaagttctgctgtatagcctgggtcgtaacccggcggtgtttgcgcgtccggaaagctaccacccgcagcgttggctggatcgtcaaggtagcggcagccgttttccgcacctggcgttcggttttggcgttcgtcagtgcctgggtcgtcgtgtggcggaagtggaaatgctgctgctgctgcaccacgtgctgaagaacttcctggttgaaaccctggagcaggaagacatcaaaatggtgtatcgttttattctgatgccgagcaccctgccgctgttcacctttcgtgcgatccaataa |
| CYP11B1 from *Ovis aries*  (CYP 11B1_Oa) | atggcgctgtgggcgaaagcgcgtgtgtggatggcgggtccgtggctgagcctgcaccgtgcgcgtccgctgggtacccgtgcgagcgcggcgccgaaagcggtgctgccgtttgaagcgatgccgcgttgcccgggtaacaagtggatgcgtgttctgcaaatctggaaagagcaaggcagcgaaaacatgcacctggacatgcaccagaccttccaagagctgggtccgatttttcgttacgacgtgggtggccgtcacatggtgtttgttatgctgccggaggatgttgagagcctgcaacaagcggaaagcctgcacccgcagcgtatgctgctggagccgtggctggcgtatcgtcaagcgcgtggtcacaagtgcggcgtgttcctgctgaacggtccgcagtggcgtctggaccgtctgcgtctgaacccggatgttctgagcctgccggcgctgcaaaagtacaccccgctggttgacggtgttgcgcgtgattttagccagaccctgaaagcgcgtgttctgcaaaacgcgcgtggcagcctgaccctggacattgcgccgagcgtgttccgttacaccattgaagcgagcaccctggttctgtatggcgagcgtctgggcctgctgacccagcaaccgaacccggatagcctgaacttcatccacgcgctggaagcgatgtttaagagcaccgtgcagctgatgttcgttccgcgtcgtctgagccgttggaccagcagcagcatgtggcgtgagcacttcgaagcgtgggattacatctttcagtatgcgaaccgtgcgatccagcgtatttaccaagaactggcgctgggtcacccgtggcactatagcggcattgtggcggagctgctgatgcgtgcggacatgaccctggataccatcaaagcgaacaccattgacctgaccgcgggtagcgttgataccaccgcgttcccgctgctgatgaccctgtttgaactggcgcgtaacccggaagtgcagcaagcgctgcgtcaggagagcctggttgcggaagcgcgtattagcgagaacccgcaacgtgcgaccaccgaactgccgctgctgcgtgcggcgctgaaggaaaccctgcgtctgtatccggtgggtatcaccctggaacgtcaggtgagcagcgacctggttctgcaaaactaccacattccggcgggtaccctggtgaaagttctgctgtatagcctgggccgtaacccggcggtttttgcgcgtccggagagctaccacccgcagcgttggctggatcgtcaaggtagcggcagccgtttcccgcacctggcgttcggttttggcatgcgtcagtgcctgggtcgtcgtgtggcggaagtggaaatgctgctgctgctgcaccacgtgctgaagaactttctggttgaaaccctggcgcaggaagacatcaaaatggtgtatcgtttcattctgatgccgagcaccctgccgctgttcacctttggcgcgatccaataa |
| CYP11B1 from *Sarcophilus harrisii*  (CYP 11B1_Sh) | atgaagcaggtgaccgagctgggtagccagctgctgcaaaccctgtggtggcgtctgggtggctgccgtggcctgaccacccgtgcggtgcaagagagcctgcgtgagagcgaaccgctgctgggtagcgttcgtccgttcgaagcgatcccgcagagccaaagcaacccgtggaccaagatgctgcgtatttggaaagacaacggctacgagaacctgcacctggaaacccaaaagaactttcagaaactgggtccgatctatcgtgacaagatgggcaccctgagcaccgttcacatcattatgccgcaggatgtggagaaggttatgaaagcggaaggccagttcccgcaacgtaaccagatgatcccgtggctgaaacaccgtcaaagccgtaagctgaaatacggtattttcctgctgaacggcaaggagtggtttaacgaccgtgtgaaactgaaccaagaagttctgagcctgcgtagcaccggccagtatatcccgttcctgaacagcgtgtgccaagattttgttaaatgcctgaacaaccagatccaaaagaacgtgcgtaaaagcctgaccttcaacatttgcccgtacgtgttccgttttacgatggaggcgggtacctacgttctgtatggtgaacgtctgggcctgctgagcaacaacccgaacccggagggcctgcgttttatccaggcgatgagcaacatgctgagcagcaccagcctgctgctgtacaccccgctggttctgagccagctgatcaacagcaagctgtggaaactgcacctggaaagctgggacgatattttccaatacgcggacaagtgcatccaaaaaatttatcaggagatgtgcctgaacggtagcccgcagtatagcggcattatggcggaactgctggcgcgtgcggacctgagcctggatgcgatcaaggtgaacattaccgagctgaccgcgggtagcgtggaaaccaccgcgttcccgctggttgcgaccctgtttgagctggcgcgtaaccaagacctgcaaagcgcgctgcgtgcggaaaccaaagaggcggaaacccgtctgaaagagcagccgcaactgctggttaaggaactgccgctgctgcgtgcgagcatcaaagaaaccctgcgtctgtacccggtgggcagcgttattcaccgttatctggcgaaagacaccgtgctgcaaaactaccacgttccggcgggtaccctgatcgagattagcctgtatgcgatgggccgtagcccggagatcttcgtgcgtccggaacgttatgatccgagccgttggctggcgccgagcagcgaacaaagcaacaaccagattagcaacttccgttttctgttctttggttttggcatccgtcagtgcattggtcgtcgtctggcggagagcgaaatgctgctgctgctgcaccacatcctgaagaacttccacgtggaaaccctgtgcaaagacaacctggatctgacctatcgttttgttctgcacccgaagagcttcccgctgtttaccctgcgtgaactgaactaa |
| CYP11B1 from *Pteropus Alecto*  (CYP 11B1_Pa) | atggcgctgcgtgcgcgtgcgggtgtttggcgtgcggcgccgcgtctgagcccgggttgggcgcgtgcggttggtgcggcggcggtggcggcgccgaaagcggtgctgccgtttgaggcgatcccgcgttgcccgggtaacaaatgggttcgtgcgctgcaagtgtggcgtgagcaaggcttcgaagacctgcacctgaagatgcagcaaaccttccaggaactgggtccgatctttcgttacgatgttggtggcaaacacattgtgtatgttaccctgccgcaagacgtggagcgtctgcaacaagcggatagctgccagccgcaacgtaccgttctggaaccgtggctggcgtaccgtcagcaacgtggtcacaagtgcggcgtgtttctgctgaacggtccggagtggcgttgggaccgtctgcgtctgaacccggatatgctgagcccgcaggcggttcaaaagtatatcccgatggtggacaaagttgcgcgtgatttcagcgcggttctgatgagccgtgtgctgcaaaacgcgcgtggtagcctgaccctggacatccaaccgagcatttttcgttacaccatcgaggcgagcaacctggtgctgttcggcgaacagctgggtctgctgggccgtagcccgagcccggcgagcctgaagttcattcacgcgctggaggcgatgtttaaaagcaccgttcagctgaccttcatgccgcgtagcctgagccgttggaccagcccgaaagtgtggcgtaaacacttcgaagcgtgggattacatctttcagtatgcgaacaccagcatccagaagatttaccaagagctggcgctgggtcgtccgcgtcagtatagcggcattgttgcggaactgctgctgcaagcggacctgaccctggatagcatcaaagcgaacagcattgacctgaccgcgggtagcgtggataccaccgcgtatccgctgatgatgaccctgtttgagctggcgcgtaacccggaagttcagcaagcgctgcgtcaggaaaccctgcgtgcgaaggtggcgatcagcgacaacccgcaacgtgcgctgaccgagctgccgctgctgcgtgcggcgctgaaagaaaccctgcgtctgtacccggtgggtatgaccgttgagcgtcaagtgggcagcgatctggttctgcaaaactatcacattccggcgggtacgatggtgtacgttcagctgtattgcctgggccgtaacccggcggtgtttacccgtccggaacgttaccacccgcaacgttggctggacaaccgtggtagcagcacccgtttcccgtatctgagcttcggttttggtccgcgtcagtgcctgggccgtcgtctggcggaaaccgaaatgctgctgctgctgcaccacgttctgaaaaacttccaagtggaaaccctgacccgtgaagatgttcgtatgatctaccgttttattctgatgccgagcaccctgccgctgctgacctttcgtgcgctggacgcgctgccgggtcagccgcgtgatcaaaccagcagcccgtaa |
| CYP11B1 from *Coprinopsis cinerea okayama*  (CYP 11B1_Cco) | atggcgagcaacgcgctgctggttgtggcgctgaccgttaccgtggttctggtgctgtaccgtgcgctgtataacctgttcctgagcccgctgagcgcgatcccgggtccgtggtacgcggcgattagcaacatctggattggctatcaccaactgtgcctgcgtcagtgcaagaccgttcaaagcctgtttgatacctacggtccggtggttcgtatcggcccgaacaaagtggcgttctgcgatgcgagcgcgatgcgtgcggtttacagcgtgtataagtttgacaaaagcacctactataagggtctggttaccatgaccaccctggaccacgcgacctatagcatccgtcgtaaggcgtacagcccgcactataccccgccgaacattgcgaaattccagccggaagtgcacgaatttaccctgcaactggtggatatcctggagaacattgcgggcaaggcgagcctggaatgcatggcgctgttccgtaacctgatggttgacgtgatgaccgaaaccctgtttggctaccgtctgggtgcgctgggcaaatgggcgatggatgcggaagacccgctgagcaccgcgatcaacgatttcccgaagcgtggcatcctgcgtagcgttattccgagctgggtgtggaaactgatctgccgtgtgccgaacaaccgttggcgtcagtggtgcgacagcgataacattatggcggagatgaacgcgggtagccacggcgagccggaacgtccgaccctgctgcaacgtctgctgcaacaccgttacagcgcgaagcagccggttccggactgcgatatcattagcgagtgcatgggtcacctgatcgcgggcgcggataccagcagcaccaccatcagctatattctgtgggaactgagccgtcgtccggacattgtggcgaagctgcaagcggagctggatgaagcgatcccggacagccgtgcgattccggacatcgcgattctgcaagagctgccgtacctgaacggtctgatcaaggaagcgctgcgtctgtacaccgcggcgccgagcctgctggagcgtgtggttccgagcagcagcagcaaaccgaaccaccaggacgaagattttgacctgctgggttatgcgctgccggcgggtaccattgttagcacccaagcgtggagcatgcaccgtaacccggcggtgtatccgagcccggagagctttctgccggaacgttggctggagagcagcagcgcgagcccggatcagctgttccaaatgcaccagcacctgatggcgtttggtgcgggcgttcgtgtgtgcggtggccaaaacctggcgcagatcgttctgcgtgtggttgtggcgatgattgttcgtaacttcaacgtgtttgcgccggaggaaaccaacgagcgtagcatggaaatcaaagacagcttcgtgatttttccggcggcgatggaatgcaagctgatctttaaaccgcgtcagcaataa |
| CYP11B1 from *Magnaporthe oryzae*  (CYP 11B1_Mo) | atgctgaacatgagcttcagcgcgggtagcagcagcagctgcgcgatgctggcgaccgcggcgatctggggtattagcgcggtggttgcgtacgcggtgtttgcgcgttttcgtgattggcgtcgtctgaaacatattgatggtccgagcctggcgggttggaccgattactggatgattcgtagccagctgagcggtcgtatgaacctggatctggcggacgtggttcgtgagtatggtccggtggcgcgtatcggcaccaacaccattgtgtgcgcggacgttaaggaactgcgtaaaatctgggcggttcgtagcacctggaaacgtccgcgttggtacctgggtctgcgtattgatccgtatagcgacaacgtgttcagcctgatggacgataaagttcacgaaaccctgcgtagcaagctgatgccgggttacggtggcaaagatgtggacggcgttcacgaaatcattgatgagcagatcagcctgtttgtgcgtctgctggaggacaagtatctgagcgttgatggcaaggacagcgaaggcaagagcttcctgccggtggatctggcgcgtaaagttcaatttctgaccctggacatcattagcagcctggcgttcggcgagagctttggcaacctggtggcggacgatgacgcgctgggttacatcagcaccaccgaaaagagcatgccgatgctgatgttcgtgaccctggttccgtggctgaccgcggtttggcagagcccgcgtctgcgttgggcgtttccggatgcgcgtaaaatggtgggtatcggcgacgttatggcgattgcgcgtcgtgtggttggtgaacgttatggcgaaaagccggaagtggttaaacgtgatatgctgggtagcttcgtggcgcacggtctgaccaagggtgaagcggaaagcgagacgatggtgcaaattgttgcgggcagcgacaccagcgcgaccgcgattcgtagcaccctgctgtttatcattaccaacccggcggtttacagccgtctgcaagcggagattgatgcggcggcggcggagggtcgtatcagcagcccgattaccgatgcggaggcgcgtgcgctgccgtacctgcaagcggtgattcgtgaaggtctgcgtatgtatccgccggcgaccggcctgctgccgaaggttagcagcaaagatgagatcctgtgcggcaaacacattccggcgggtaccgatgtgggttgggcggtgtggccggttatgcgtgatcgtggtgttttcggcgatgacgcggacctgtttcgtccggaacgttgggtggaggcggcgccggaacagtggcgtgtgatggatcaaaccgttatgatggacttcgcgaccggtagccgttgggaatgcctgggcaagaccatcgcgatgattgagctgaacaaagcgtatgttgaactgctgcgtcgtttcgatatcaccctgctggacccgagcaacccgtggcacagctttaacgcggcgttctttatccaaaagaacatgaacgtgaagattagccgtcgtaagaccccggtttaa |
| CYP11B1 from *Sorex araneus*  (CYP 11B1_Sa) | atggcgtttcgtgcgcaggtgccgctgcgtctggcgcactgctgggcgcaaaccctgggttaccgtagcgcgctggcgggtgaaccggttctgccgtttgcggcggtgccgcagtatccggttagcccgggtatgaagctgctgtggggtaaaggctttgactgggaacacctgccgctggagatgcaccgtgcgttccaagagctgggcccgatctttcgtctggatgtgggtggcaagagcaccgtgtgggttatgcagctggaggcgaccgaacgtctgaaacaagcggaaagccgtttcccgcaccgtggttttccgatgccgtggatgagccaccgtgagcagcgtggtcacaagctgggcgttttcatgctgagcggtccggaatggcgtgcgcagcgtatgagcctgaacccgaacatgctggcgctgcaagcgatttacaacttcctgccgatggtggacagcgttgcgcgtgagtttagccagaccctgcgtgcgaaggtgctgcaaaacacccgtcgttgcctgaccctggatatgaaaccgagcctgatgcgttttagcgcggaagcgagcaacctggcgctgtacggcgagcgtctgggcctgctgagcgaggcgccgaaacaggcgagcgaagacttcctgagcgcgctggaagtgatctttgaaagcaccgttgagctgatgtatcaaccgcgtagcctgagccgttggaccagcagcgcgaagtggaaagagcacttcgaagcgtgggattacatctttcactatgcgagcaacagcatgcagcgtatttaccaagagctgagcctgggtcacccgcagcactatagcggcatcgttgcggaactgctggcgaacgcggacatgaccctggataccattcgtgcgaacagcaccgacctgaccgcgggtagcgtggataccaccgcgtacccgctgctgatgaccttctttgaactggcgcgtaacccggaagtgcagcaagcgctgcgtcaggaaagcctgcaagcggaagcgaccattctggcggacccgcgtaaggcgctgagcgagctgccgctgctgcaagcggcgctgaaagaaaccctgcgtctgtacccggtgggcccgattctggaccgtgagctggcgagcgatatcgttctgcacaactactggattccgagcggtaccaccgtgcgtgttagcctgtatagcaccggccgtgatccgaccattttcgcgagcccggaacgttatcacccgcagcgttggctggaccaaaaggataccaacacccgtctgccgcgtctggcgttcggttttggcatgcgtcagtgcctgggtcgtcgtatggcggaagtggttatgctgctgctgatgcaccacgtgctgaaaaactttatggttgaaaccctgacccaagaggacctgcgtatgtgctatcgtttcatcctgattccgagcaccaccccgctgtttaccatccgtgtgattgattaa |
| Adx_4-108_ from *Bos taurus* | atgcgtctgctgcgtgttgcgagcgcggcgctgggtgataccgcgggccgttggcgtctgctgctgaagagcagccagttcattaaggtgagctgtagcggtagctggatcagcgcggcgcaacgtgcgtttatttgctacagcaagagcggcaacatcacctgcttcctgcgtagcgaggacaaaatcaccgttcactttattaaccgtgatggtgaaaccctgaccaccaagggtaaaattggcgacagcctgctggatgtggttgtgcagaacaacctggacatcgatggttttggtgcgtgcgagggtaccctga |
| AdR from *Bos taurus* | atggcgccgcgttgctggcgttggtggccgtggagcagctggacccgtacccgtctgccgccgagccgtagcattcagaacttcggtcaacactttagcacccaggagcaaaccccgcagatttgcgtggttggtagcggtccggcgggtttctacaccgcgcaacacctgctgaagcaccacagccgtgcgcacgttgacatttatgaaaaacagctggtgccgtttcgtctggtgcgtgtgtggctggcgctgaccaccccgcgtagccgtatgctgctgaacaccttcacccaaaccgcgcgtagcgaccgttgcgcgttttacggtaacgttgaggtgggtcgtgatgtgaccgttcaggaactgcgtgtttaccgtctgaccgcggtggttctgagctatggtgcggaggaccaccaagcgctggatatcccgggcgaggaactgccgggcgttttcagcgcgcgtgcgtttgtgggttggtataacggcctgccggagaaccgtgaactggcgccggacctgagctgcgataccgcggttatcctgggtcagggcaacgttgcgctggatgtggcgcgtattctgctgaccccgccggaccacctggagaagaccgatattaccgaggcggcgctgggtgcgctgcgtcaaagccgtgtgaaaaccgtttggattgttggtcgtcgtggtccgctgcaagtggcgttcaccatcaaggagctgcgtgaaatgattcaactgccgggtacccgtccgatgctggacccggcggattttctgggtctgcaagaccgtatccgtgaagcggcgcgtccgcgtaagcgtctgatggagctgctgctgcgtaccgcgaccgaaaaaccgggtgttgaggaagcggcgcgtcgtgcgagcgcgagccgtgcgtggggcctgcgtttctttcgtagcccgcagcaagttctgcgtctgccggatggtcgtgcgcgtcgtagcgcgtggcagagcccggagctggaaggtatcggtgaagcgcacccgggtagcgcgcactggggctgcggtggcccgccgtgcggtctggttctgagcagcatcggctacaagagccgtccgattgacccgagcgtgccgttcgatccgaaactgggtgtggttccgaacatggaaggccgtgtggttgacgttccgggtctgtattgcagcggttgggttaagcgtggtccgaccggcgtgattaccaccaccatgaccgacagctttctgaccggtcagatcctgctgcaagatctgaaagcgggtcacctgccgagcggtccgcgtccgggcagcgcgttcatcaaagcgctgctggacagccgtggtgtttggccggtgagctttagcgactgggagaaactggatgcggaggaagtgagccgtggtcaggcgagcggcaagccgcgtgagaagctgctggacccgcaggaaatgctgcgtctgctgggccactaa |

**
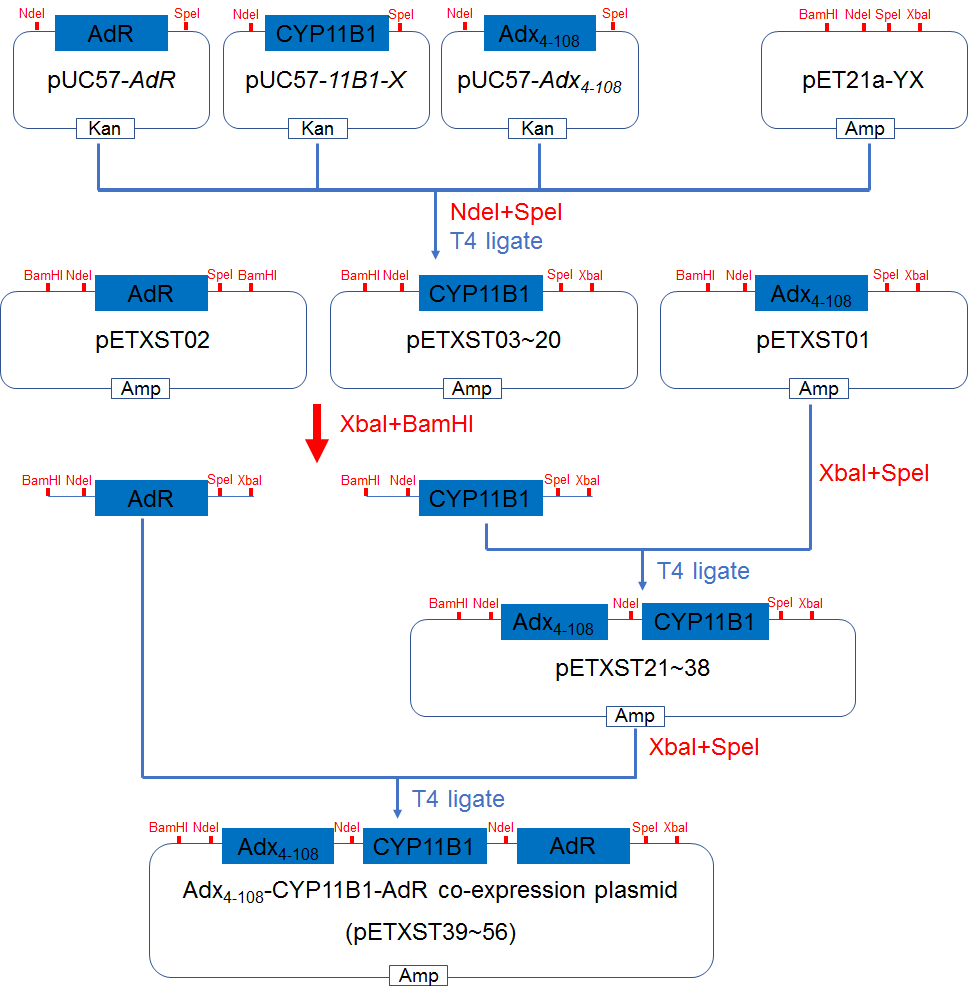
**

**Figure S1.** Schematic representation of the constructing strategies for Adx_4-108_-CYP11B1-AdR co-expression plasmids (pETXST39~56).

**
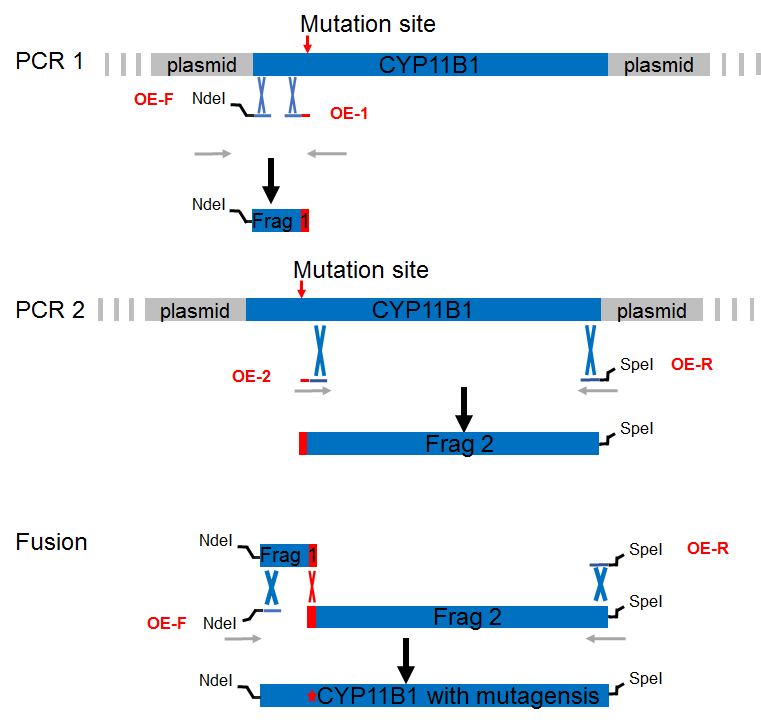
**

**Figure S2.** Schematic representation of the mutagenesis strategy applied in this study. The star marks the mutagenesis sites, while the arrows with the same color represent one pair of primers used in the PCR amplification.


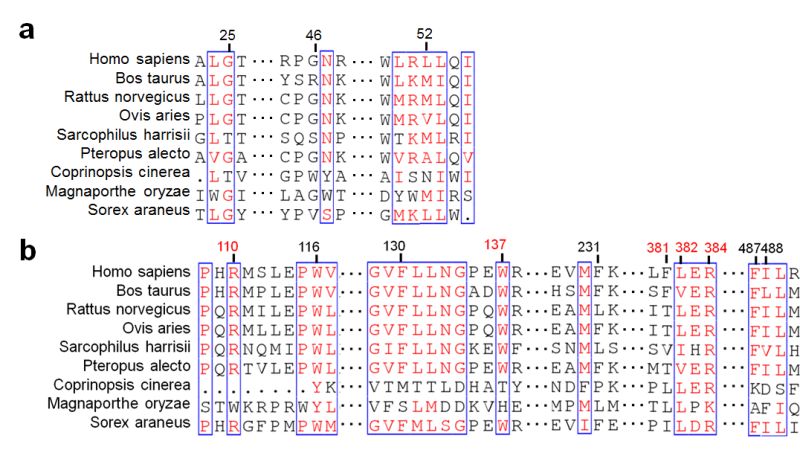


**Figure S3.** Alignment of CYP11B1s from nine species screened in this study. (**a**) is used to identify mutants equal to those reported in *H. sapiens.* In (**b**), red numbers represent the sites of the amino acids interacting with heme, while black number represent the sites of the amino acids interacting with the substrate.

**
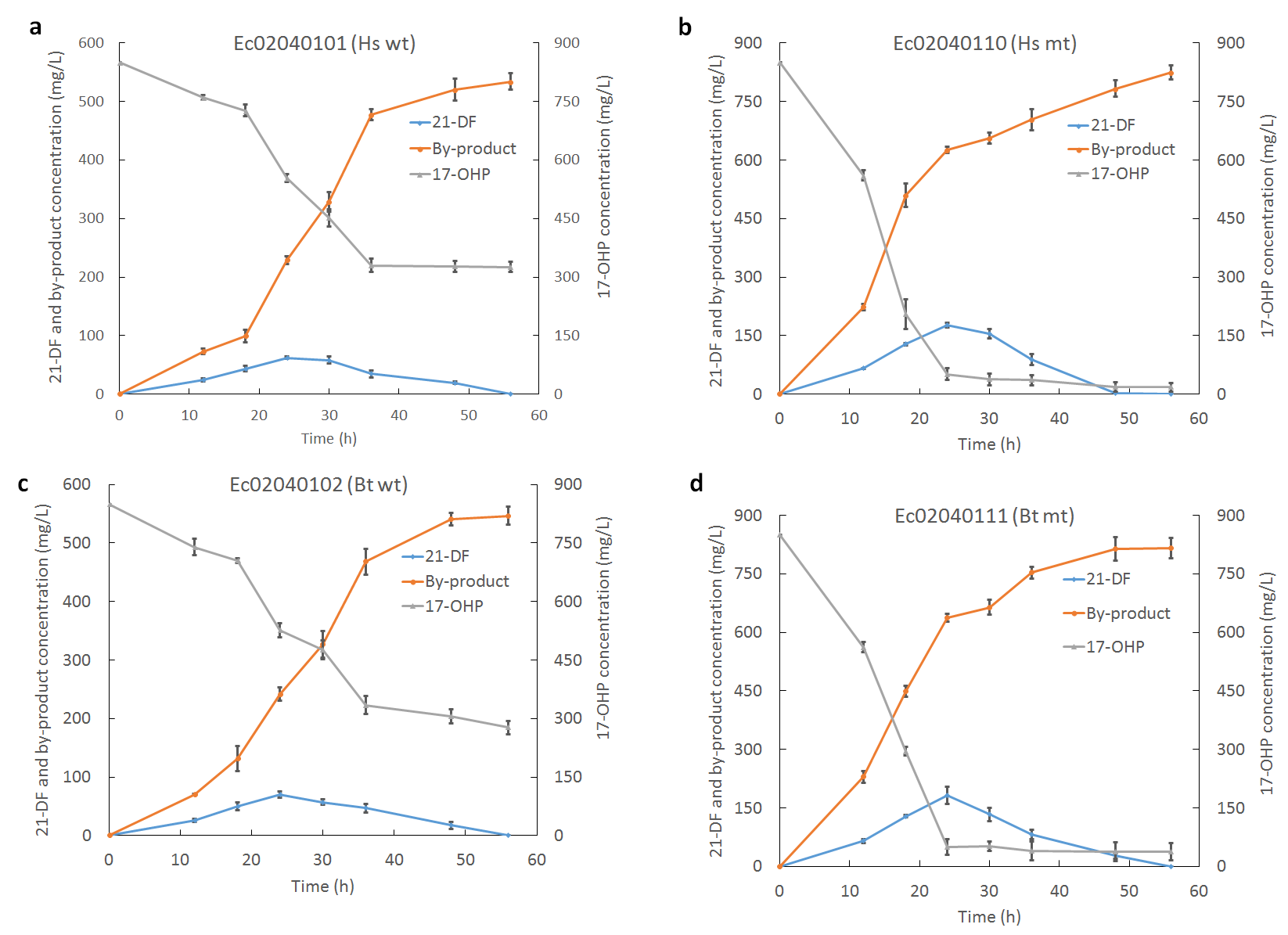
**

**
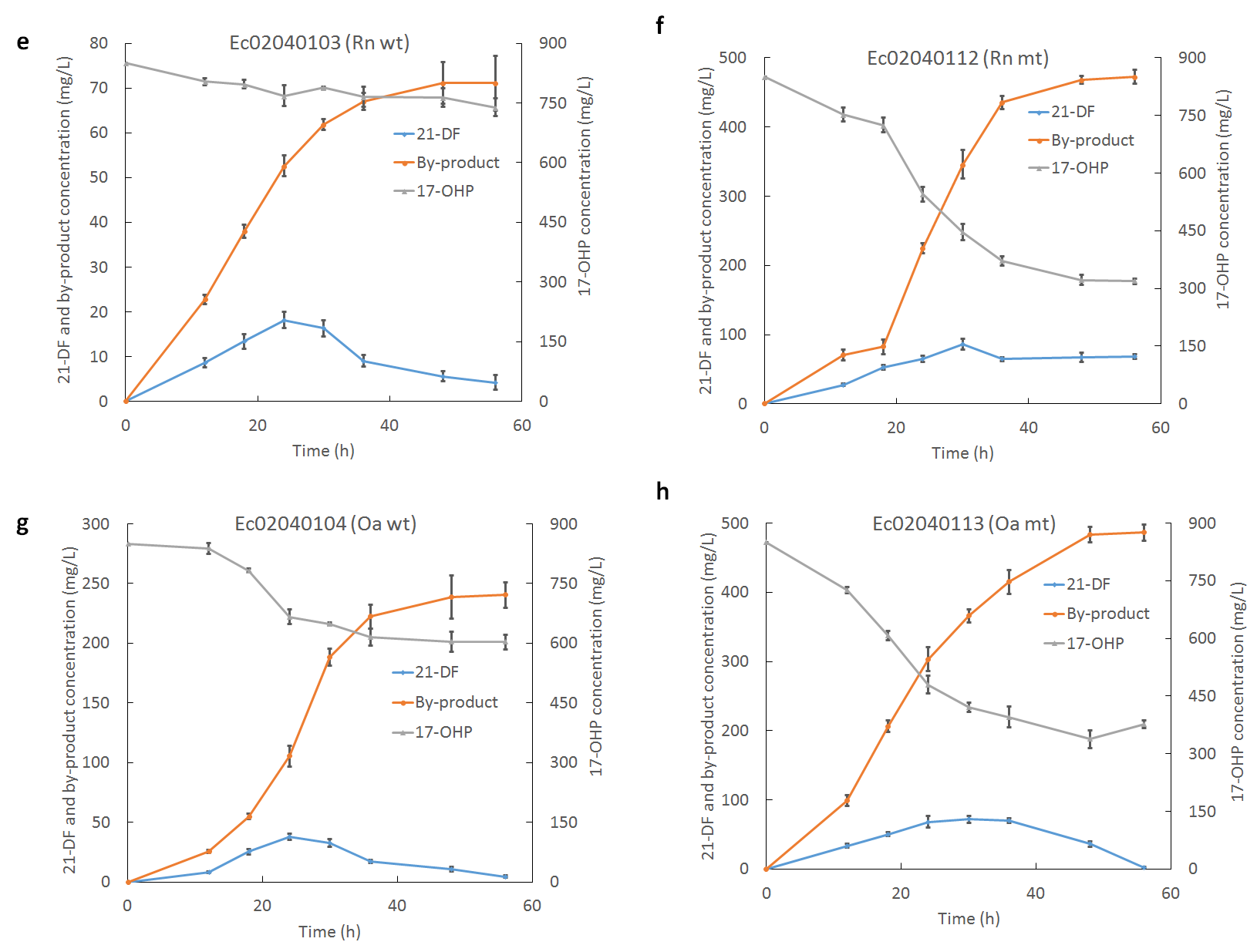
**

**
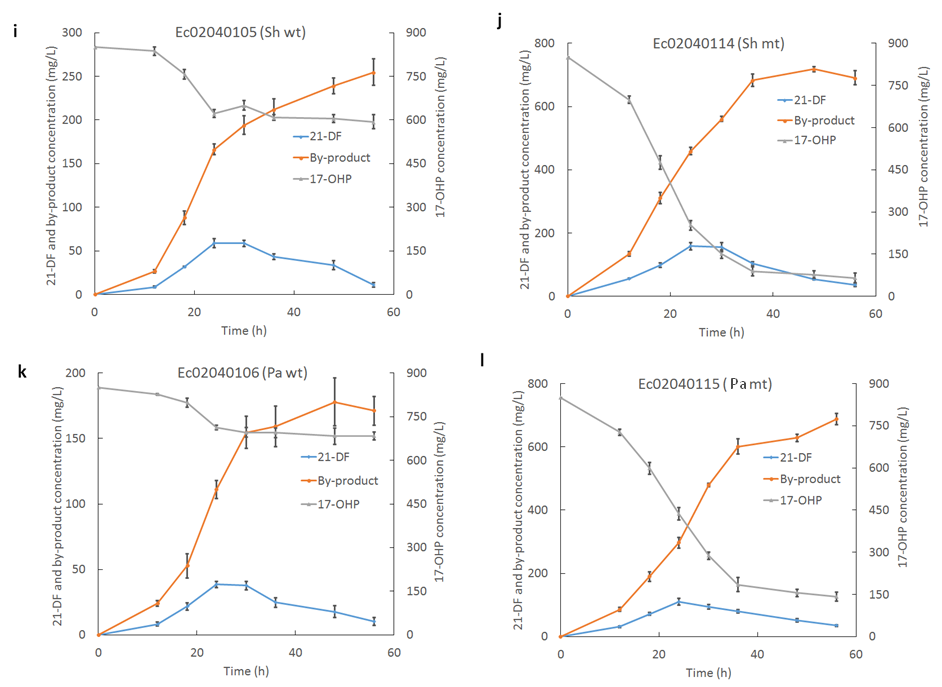
**

**
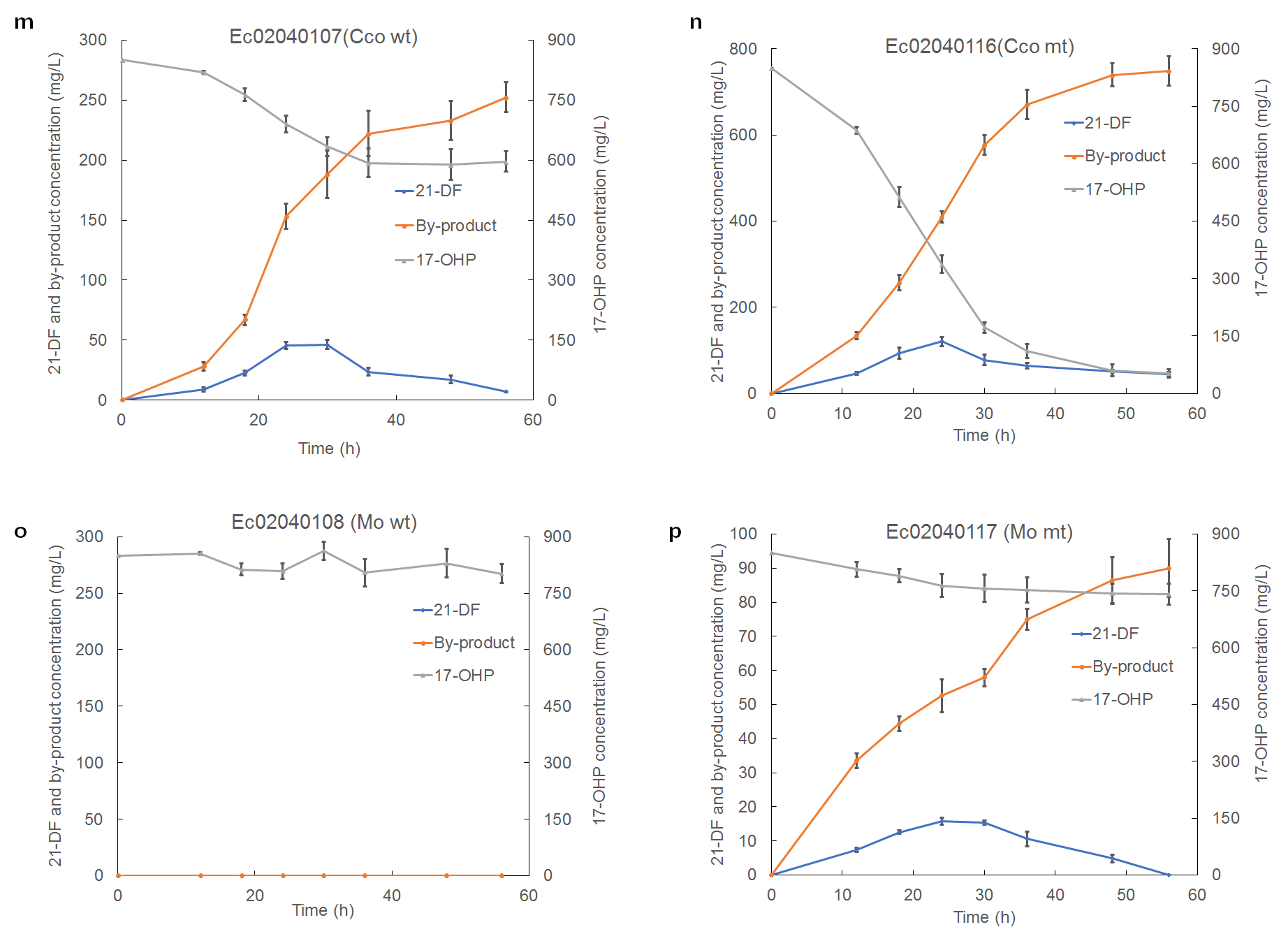
**

**
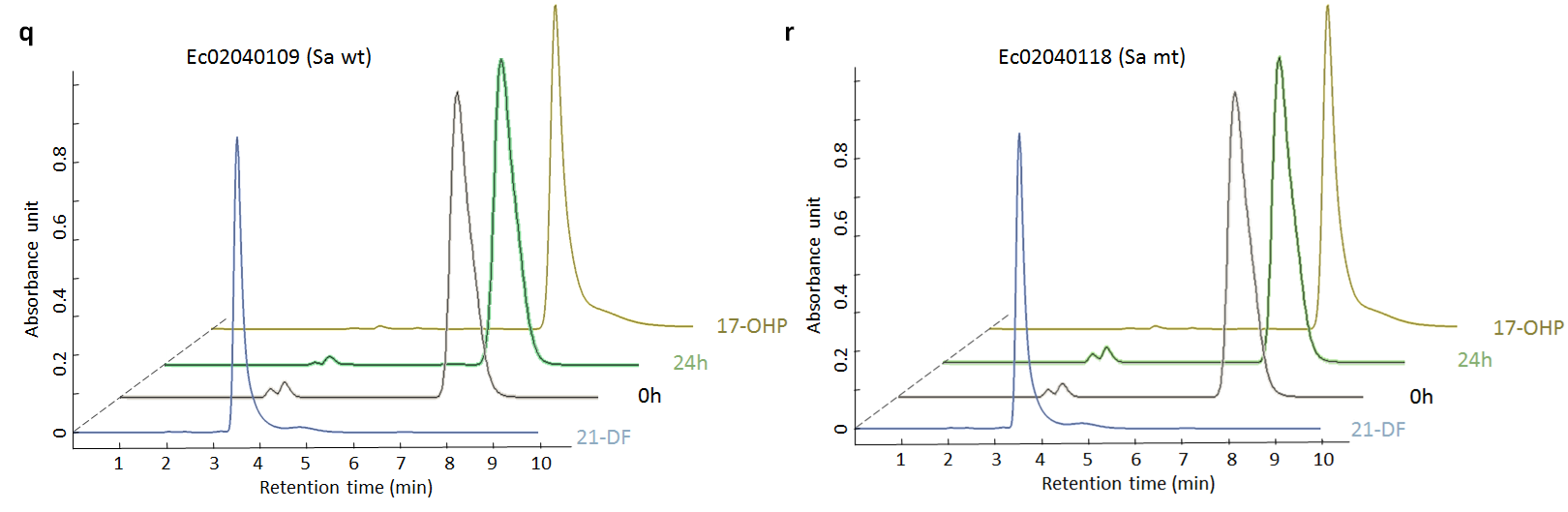
**

**Figure S4.** Conversion of 17-OHP to 21-DF by biocatalysts harboring different wild-type and mutated CYP11B1s from diversity species. (**a-p**): 21-DF, by-product and 17-OHP concentrations at different time point. (**q-r**): Products of strains Ec02040109 and Ec02040118 at 0 h and 24 h analyzed by HPLC. Since these two strains could not achieve our desired conversion, further detailed experiences to take examples at different time points were not further conducted. Ho, *Homo sapiens*; Bt, *Bos taurus*; Oa, *Ovis aries*; Sh, *Sarcophilus harrisii*; Pa, *Pteropus Alecto*; Cco, *Coprinopsis cinerea okayama*; Mo, *Magnaporthe oryzae*, Sa, *Sorex araneus*. wt, wild-type; mt, mutated enzyme.


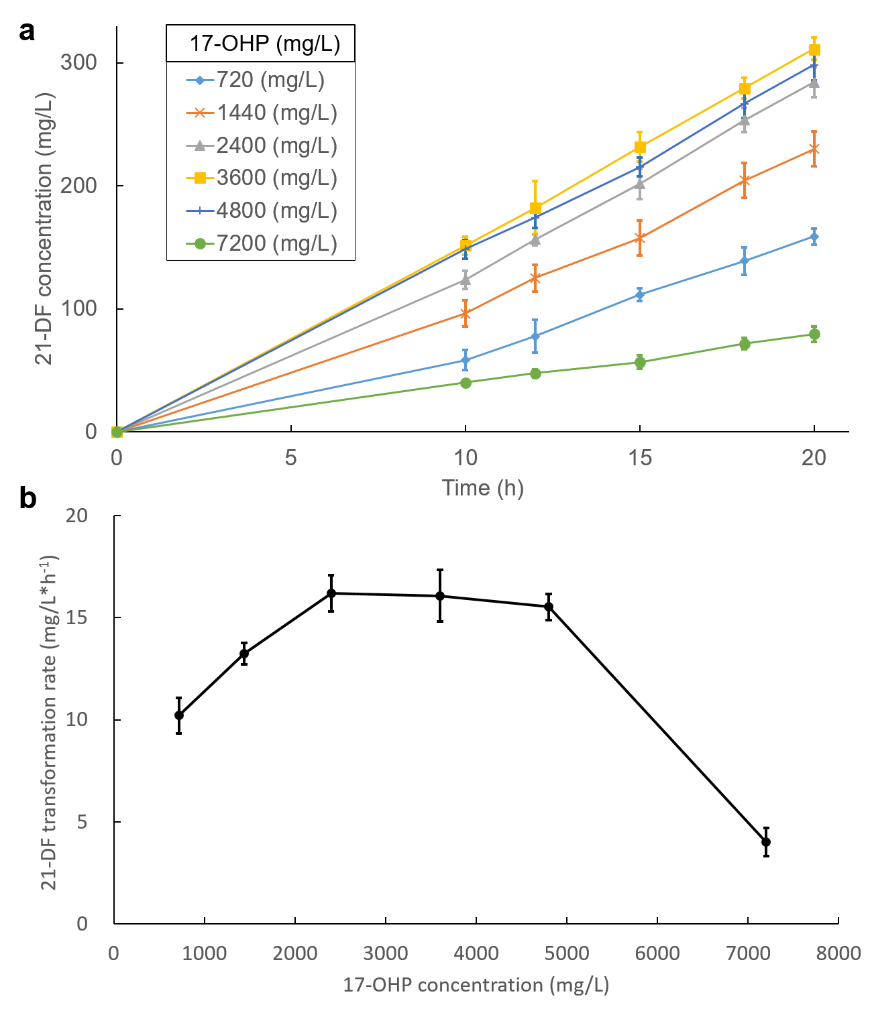


**Figure S5.** Optimizing substrate (17-OHP) concentration for higher biocatalyst efficiency. Steroids conversion was performed by resting cells in potassium phosphate buffer. 17-OHP were supplemented into the buffer at very beginning of the biotransformation with concentrations of 720 mg/L, 1440 mg/L, 2400 mg/L, 3600 mg/L and 7200 mg/L. Samples were taken at 10h, 12h, 15h, 18h and 20h (**a**). And the 21-DF transformation rate under each substrate concentration were illustrated in (**b**).

**
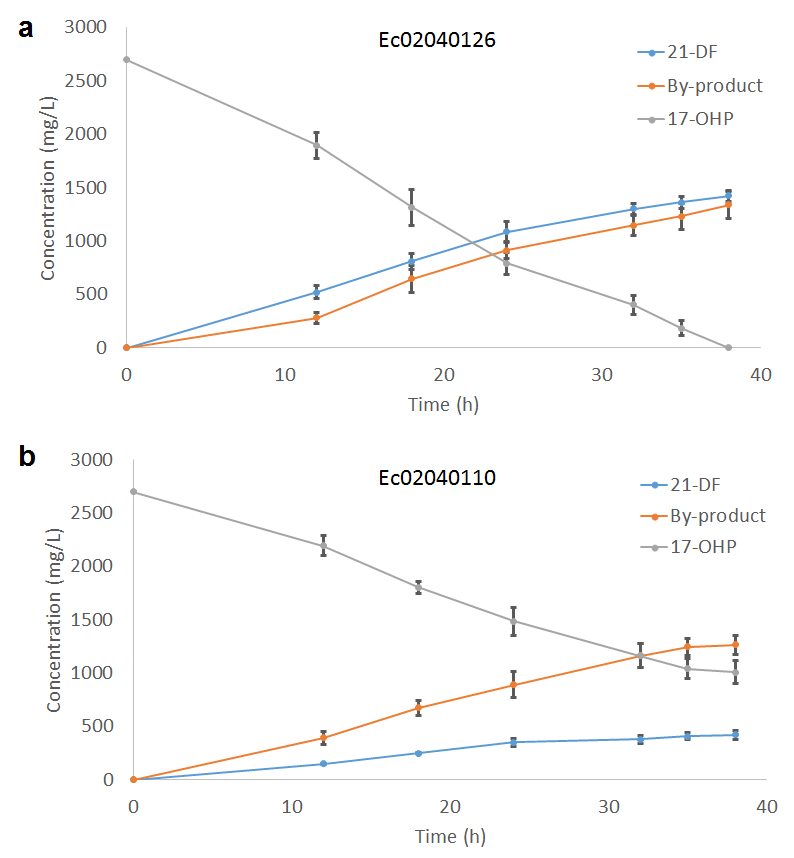
**

**Figure S6.** Biotransformation of 17-OHP to 21-DF by strains Ec02040126 (**a**, harboring CYP11B1_Hs mutant G25R/G46R/L52M/F381A/382S/I488L) and Ec02040110 (**b**, the control strain in Fig. 4d, harboring CYP11B1_Hs mutant G25R/G46R/L52M) during the time course.
